# MagCluster-GMAF: A Physics-Inspired Hybrid Graph Architecture for Long-Range Magnetic Property Prediction

**DOI:** 10.3390/nano16211422

**Published:** 2026-11-04

**Authors:** Yu Zhang, Jiale Wang, Haotian Liu, Yishuo Wang, Guangjie Liu, Wei Liu

**Affiliations:** 1 School of Artificial intelligence, Changchun Normal University, Changchun 130032, China; wangjialehhh@gmail.com (J.W.); qx202411023@stu.ccsfu.edu.cn (H.L.); 15152117649@163.com (Y.W.); 2 iFLYTEK Co., Ltd., No. 666 Wangjiang West Road, Hefei 230088, China

**Keywords:** MagCluster-GMAF, long-range magnetic property prediction, physics-inspired hybrid graph architecture, Mamba-2, two-dimensional doped magnetic nanomaterials

## Abstract

Understanding and predicting the structure–property relationships in magnetic clusters remain challenging due to the complex interplay between geometry, electronic structure and spin configurations: density functional theory suffers from prohibitive computational cost for large-scale exploration, while existing graph neural networks struggle to balance long-range magnetic correlation capture, topological fidelity and scalability. We present MagCluster-GMAF, a physics-inspired hybrid graph architecture with magnetic cluster-aware serialization to preserve topological and electronic similarity during graph-to-sequence transformation, paired with a dual-branch design combining a Mamba-2 backbone for efficient long-range spin correlation modeling and a lightweight attention module as topological compensation. On a 2D doped magnetic material dataset, it achieved a total magnetic moment prediction MAE of 0.2683 μB, reducing the prediction error by 9.5% relative to the GPS + Transformer baseline and outperforming Graph-Mamba; at the 1400-node scale, it cut the GPU memory consumption by over 70% versus standard graph transformers while exhibiting near-linear empirical scaling at the studied graph scales. SHAP-based feature attribution analysis further identifies the key physicochemical descriptors governing the predictions, providing an effective approach for nanoscale magnetic material screening and the high-throughput screening of spintronic materials.

## 1. Introduction

Magnetic phenomena in low-dimensional materials, particularly doped two-dimensional (2D) systems, underpin a wide range of emerging technologies including spintronics and quantum information processing [1,2]. A central challenge in this domain is the accurate prediction of macroscopic magnetic properties from atomistic configurations, where collective behavior arises from delocalized long-range spin correlations mediated by various exchange coupling mechanisms [3,4,5]. These interactions define non-local correlation pathways that extend across multiple lattice sites and are highly sensitive to both electronic structure and crystal topology [6,7].

Despite the success of density functional theory (DFT), its unfavorable scaling severely limits its applicability for the large-scale exploration of doped material spaces. Data-driven approaches based on graph neural networks (GNNs) have emerged as a promising alternative by representing crystal structures as graphs [8,9,10]. However, conventional message-passing GNNs are intrinsically local, making them insufficient for capturing long-range magnetic correlations [11,12]. Global attention-based architectures partially address this limitation by enabling all-to-all interactions, yet their quadratic complexity renders them impractical for large supercells and high-throughput screening [13,14].

Recent advances in structured state space models (SSMs), exemplified by the Mamba architecture, provide a compelling pathway toward linear-complexity long-range modeling [15,16]. Nevertheless, applying sequence-based models to non-Euclidean crystal graphs introduces a critical and largely overlooked challenge: the loss of physically meaningful interaction topology during graph-to-sequence transformation. Existing serialization strategies, typically based on heuristic node ordering, fail to preserve the continuity of long-range magnetic interaction pathways, leading to fragmented representations that obscure the underlying physics. Therefore, the core challenge is not merely achieving efficient long-range modeling, but preserving physically meaningful magnetic interaction pathways under scalable representations. To date, no existing framework simultaneously satisfies these two requirements: global attention preserves topology but is computationally prohibitive, while linear models achieve scalability at the cost of physical fidelity.

In this work, we propose MagCluster-GMAF, a physics-informed hybrid graph architecture designed to address this fundamental limitation. The key idea is to reformulate graph serialization as an interaction-path preserving transformation, in which nodes are reorganized according to magnetic cluster affinity derived from both topological and electronic descriptors. This strategy minimizes the disruption of physically relevant localities in the resulting sequence representation. Building upon this representation, we introduce a dual-branch processing paradigm that integrates a Mamba-2 backbone with a lightweight attention module. Rather than serving as a generic global aggregator, the attention branch acts as a lightweight topological compensation mechanism that models non-local structural dependencies that are otherwise difficult to recover from the serialized sequence. This design establishes a controlled information bottleneck, where efficient long-range propagation and high-fidelity topological correction are decoupled yet synergistically integrated.

Extensive experiments on doped 2D magnetic systems and long-range graph benchmarks demonstrate that MagCluster-GMAF achieves superior prediction accuracy with low MAE while retaining near-linear empirical scaling at the studied graph scales. Beyond prediction accuracy, the proposed framework integrates efficient long-range sequence modeling with lightweight graph-structure compensation, offering a scalable approach for high-throughput magnetic materials screening. To realize this scalable modeling framework, we make three key technical contributions.

### 1.1. Interaction-Path Preserving Graph Serialization

We identify the loss of physically meaningful long-range interaction pathways during graph-to-sequence transformation as a fundamental bottleneck in applying linear-complexity models to magnetic materials. To address this, we propose a magnetic cluster-aware ordering strategy that preserves both topological structure and electronic similarity, effectively maintaining magnetic homophily in the serialized representation.

### 1.2. Physics-Informed Dual-Branch Architecture with Topological Compensation

We introduce a hybrid framework that combines a Mamba-2 backbone for efficient long-range propagation with a lightweight attention module that models non-local structural dependencies beyond the reach of one-dimensional sequence scanning. This establishes a “backbone–compensation” mechanism that balances scalability and topological fidelity.

### 1.3. Scalable and Interpretable Modeling of Long-Range Magnetic Systems

We demonstrate that the proposed architecture achieves the best MAE and MSE among the evaluated baselines on magnetic material prediction and competitive performance on long-range graph benchmarks, while providing feature-level interpretability through SHAP attribution analysis, incorporating domain priors from condensed matter physics into data-driven modeling.

## 2. Methods

Before detailing the MagCluster-GMAF architecture and experimental validation, we first define the task scope and key terminology to avoid ambiguity in the subsequent analysis. The core task of this work is to predict the total magnetic moment of doped supercells, formulated as a graph-level regression problem. This study does not seek to predict, identify, or reconstruct individual magnetic interaction pathways (e.g., pairwise exchange couplings). In particular, the term “long-range dependencies” used throughout the text refers to the architectural capacity to propagate information across the structure, not a claim of verified physical interaction mechanisms.

### 2.1. MagCluster-GMAF Model Architecture

**Figure 1:**
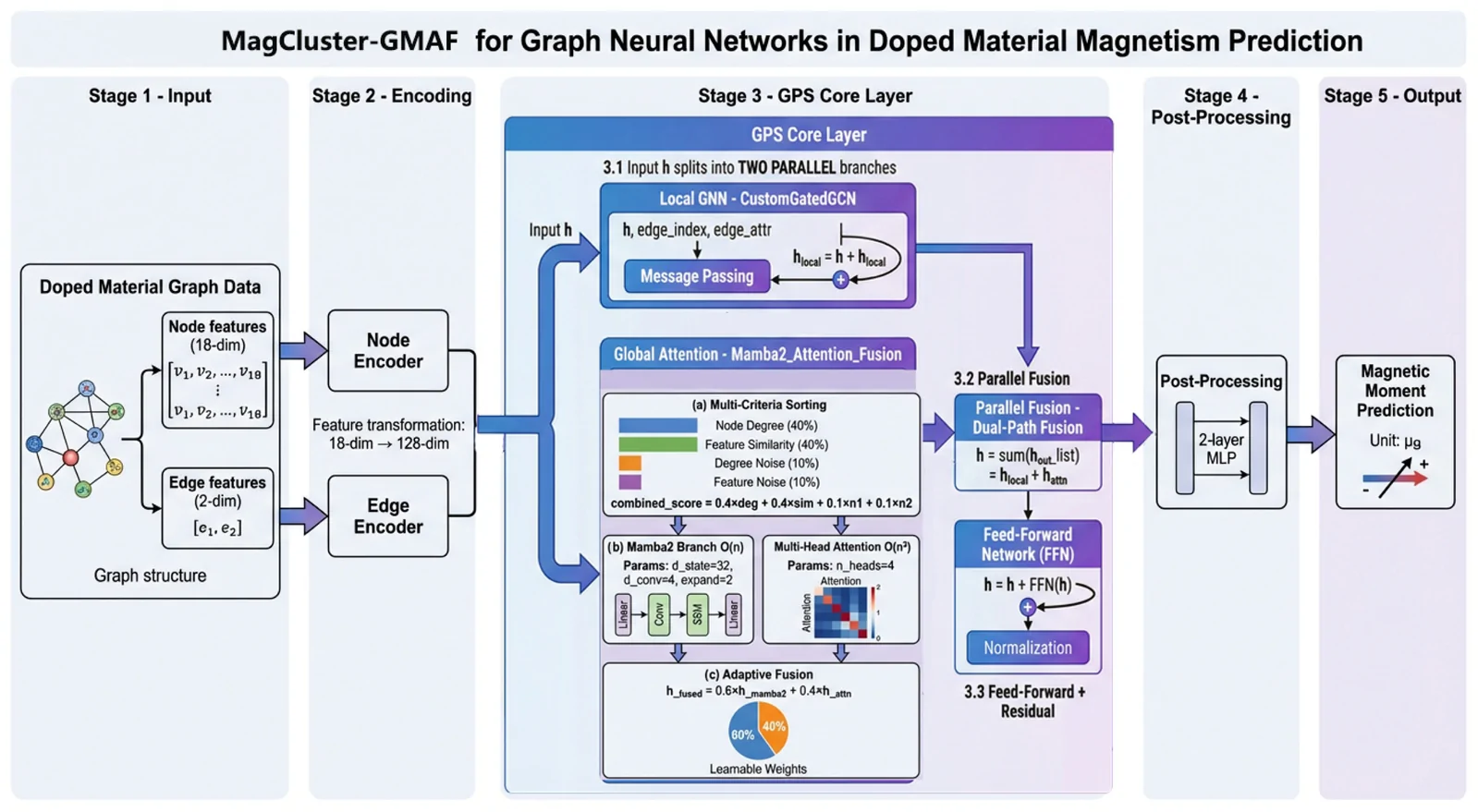
Overall architecture of MagCluster-GMAF. The diagram illustrates the end-to-end pipeline, from physics-informed property graph construction and magnetic cluster-aware sorting to the parallel Mamba-2 and attention dual-branch processing, adaptive fusion, and global magnetic moment prediction.

Overview of the Overall Architecture: In the task of predicting the magnetic properties of two-dimensional doped nanomaterials, it is crucial to accurately characterize delocalized long-range spin correlations across the crystal lattice [17]. However, traditional global graph learning models face severe computational scalability bottlenecks. To break the trade-off between computational efficiency and physical expressiveness, this paper proposes a novel hybrid graph neural network architecture—MagCluster-GMAF. As shown in Figure 1, this architecture enables end-to-end magnetic moment prediction, with its processing pipeline primarily consisting of the following five core modules:

(1) Physics-Informed Graph Construction and Shallow Encoding

Given a property graph 𝒢 = (𝒱,ℰ) of a doped system, where V is the set of atomic nodes and ℰ is the set of edges representing chemical bonds and nearest-neighbor interactions, to fully enable the model to capture the magnetic essence of the material, we embed the nodes and edges into a feature space with strong physical prior knowledge: for each atomic node, we extract 18-dimensional composite features (encompassing electronic structure characteristics such as elemental arrangement, number of unpaired electrons, and number of electrons in d/f orbitals, as well as explicit magnetic indicators such as theoretical magnetic moment and spin state); for each edge, we extract 2-dimensional distance features (absolute distance and inverse distance) to precisely characterize the spatial decay of exchange coupling strength. Subsequently, the raw physical features are uniformly mapped via independent linear encoders to a high-dimensional latent representation space of dimension d, H^(0)^ ∈ ℝ^|𝒱|×d^ (d = 128), providing a solid semantic foundation for subsequent long-sequence modeling.

(2) Magnetic Cluster-Aware Sorting & Flattening

Since the standard Mamba architecture cannot directly process non-Euclidean, unordered graph structures, the high-dimensional graph topology must be reduced to a one-dimensional sequence. Unlike traditional Graph-Mamba [18], which relies on a single-node-degree heuristic for dimensionality reduction, this module innovatively introduces a physically-inspired multi-hybrid node sorting strategy. This strategy combines topological centrality (node degree, accounting for 40%) with microscopic physical semantic similarity (accounting for 40%) according to specific weights, and incorporates moderate regularization perturbations (degree and feature noise each accounting for 10%). Driven by feature weights, this ordering mechanism forces atoms with similar physical properties closer together in the downsampled sequence, implicitly simulating “magnetic clusters” within the material. After rearrangement, the discrete graph is converted into a regular dense tensor sequence, and the framework generates a corresponding padding mask to define the effective boundaries of the supercell. This design effectively suppresses the model’s overfitting to specific node arrangements while preserving local homogeneity.

(3) Mamba-2 and Attention-Based Parallel Cooperative Processing

After serialization and masking, the feature tensors are simultaneously fed into the core of this architecture: the dual-branch parallel collaboration module. Main Branch (Mamba-2 Branch): Serving as the primary feature extractor for capturing global spin correlations, it is configured with the state dimension d_state_, the local convolution kernel d_conv_, and the feature expansion factor E. This branch performs state-space scans over the sequence with a strict O(n) linear time complexity, efficiently modeling long-range dependencies across the serialized atomic sequence. Auxiliary Branch (Attention Branch): Acting as a lightweight “Topological Regularizer”, this branch is configured with four attention heads (Multi-Head Attention). Although it has an O(n^2^), its actual computational overhead is minimal within the feature-dimension-reduced subspace. Its core objective is to establish fully connected global routing, capturing non-local dependencies and doping-induced distant structural distortions that are difficult for a one-dimensional scan to cover.

(4) Task-Adaptive Dynamic Fusion

To deeply align the two manifolds described above—which possess different receptive fields and spatial induction biases—the model employs a dynamic soft-routing mechanism based on Softmax normalization. We introduce a set of learnable fusion weight parameters (empirically initialized with 60% from the Mamba-2 branch and 40% from the Attention branch). During each forward pass, this mechanism adaptively adjusts the linear superposition ratio of the two branch feature outputs based on the topological complexity of the current input doped system. This dynamic weighting endows the hybrid architecture with improved task adaptability and generalization robustness.

(5) Global Readout and Graph-Level Prediction

After feature fusion, the implicit representations at the node level are refined non-linearly through residual connections, one-dimensional batch normalization (BatchNorm1d), and a feedforward neural network (FFN). Subsequently, a global mean pooling mechanism is employed to aggregate all atomic features indiscriminately into a macroscopic graph-level representation of the entire material system. Finally, a prediction head composed of a multi-layer perceptron (MLP) projects the high-dimensional graph features into a scalar output $\hat{y}$, which represents the model’s predicted total magnetic moment of the system (μ_B_).

We compare this model with several state-of-the-art baseline models in the field of graph learning and conduct an in-depth analysis in terms of prediction accuracy, computational complexity, and architectural ablation studies. To comprehensively evaluate the model’s performance, we selected multiple graph datasets spanning different domains and scales for this experiment, with magnetic property prediction for two-dimensional nanomaterials (CMR Impurities) serving as the core task. Additionally, we conducted benchmarking using four dataset pairs from the Long-Range Graph Benchmark (LRGB) [19] and the GNN Benchmark [20] to further validate the model’s ability to model long-range context and its generalizability across different topological heterogeneities and task types. To rigorously demonstrate the superiority of our architecture, as shown in Table 1, we selected five top-tier baseline models representative of the graph long-range modeling field for comprehensive comparison. These models encompass various evolutionary paradigms, including exact fully connected, approximate linear, sparse graph attention, and pure state-space models.

### 2.2. Dataset Construction and Preprocessing

The data for this study were sourced from the Computational Materials Repository (CMR). Through high-throughput first-principles calculations, this database systematically compiles the crystal structures and corresponding physicochemical properties of approximately 17,000 doped two-dimensional nanomaterials [25]. The raw data are stored in xyz file format, containing precise atomic spatial coordinates, element types, and energy and magnetic parameters derived from DFT calculations. During the data preprocessing stage, this work transforms the traditional structural description into a property graph representation. Specifically, each atom is mapped to a node v ∈ V in the graph. To accurately capture the chemical bonding relationships and delocalized long-range physical interactions within the material, we employ a method combining dynamic thresholding with hard truncation to construct the edge set ℰ. Based on the sum of interatomic distances and covalent radii, and with a global search threshold set to 5.0 Å, we ensure that the graph structure not only includes covalent bond information from first-order neighbors, but also encompasses second-order neighbors and inter-shell atomic interactions, which are crucial for magnetic correlations. Compared to the computer vision (CV) processing scheme—which projects three-dimensional structures into two-dimensional pixel images—the graph representation scheme G = (V, ℰ, X, Efeat) proposed in this section possesses significant physical advantages. The graph structure directly preserves the coordination environment and many-body interactions between atoms at the three-dimensional topological level, avoiding the sparsification of physical information caused by dimensionality reduction through projection. Regarding dataset partitioning, this study follows the principle of stratified sampling, dividing the data into training, validation, and test sets at a ratio of 7:1.5:1.5. The statistical deviation in magnetic moment distribution across each subset was strictly controlled to be less than 2%, laying a solid foundation for verifying the generalization performance of subsequent models.

### 2.3. Deep Physical Feature Engineering

The accuracy of magnetic prediction tasks essentially depends on the model’s ability to capture the microscopic states of electronic structure [26]. Since the original dataset provides only atomic positions and element symbols, relying solely on coordinates makes it difficult to capture deep physical mechanisms such as spin polarization and charge transfer. To address this, this work draws on prior knowledge from condensed matter physics and incorporates high-fidelity deep physical features into the material graph based on the periodic table, Hund’s rules, and electron configuration principles.

This model takes as input only ideal eigenstates under the “isolated atom limit”, yet predicts quantum many-body physical states resulting from coupling within a complex two-dimensional lattice environment [27]. The macroscopic total magnetic moment of a doped system is by no means a simple linear scalar sum of the localized magnetic moments of individual isolated atoms. It involves extremely complex spin frustration, charge transfer, and the cancellation of antiferromagnetic coupling across shells [28]. The model inputs the “prior state of an isolated atom”, while the prediction concerns the “macroscopic state resulting from coupling to a complex lattice environment”. When transition metal atoms with unpaired electrons are embedded into a two-dimensional material lattice, a series of dramatic microscopic state transitions are induced: the quenching and reshaping of localized magnetic moments. Strong ligand field effects and d-p orbital hybridization between the atom and the host lattice lead to significant charge transfer, causing the atom’s true localized magnetic moment within the lattice to deviate sharply from its isolated state [29]. The core challenge and motivation of this study: We endow the model with a “physical prior state of isolated atoms”, with the fundamental aim of establishing initial feature anchors that compel the MagCluster-GMAF architecture to leverage its powerful nonlinear graph topology awareness to learn the highly complex quantum many-body evolution mapping functions within real materials:

F:{*M_isolated_*,*Topology*} → *M_coupled_*

The incorporation of such physical prior knowledge reduces the blind search required in deep learning across the vast parameter space, serving as an essential foundation for achieving efficient, high-fidelity predictions of material properties.

Node Features: We designed an 18-dimensional composite feature vector for each atomic node, aimed at maximizing the encoding of quantum chemical information within a limited feature bandwidth. These features can be categorized into the following four types, with their detailed composition shown in Table 2:

Basic Structural Features (8 Dimensions): These features are designed to identify the fundamental chemical identity of atoms. Element types and their valence electron configurations are distinguished by normalizing atomic number and group number; a binary “doping indicator” is introduced to mark sources of magnetic modification within the system; dedicated feature bits have been specifically designed for 3d/4d/5d transition metals and chalcogenides, as the d-orbital electron configuration of transition metals is the primary carrier of localized magnetic moments, while chalcogenides are key to constructing the framework of two-dimensional materials.

Periodic Table Properties (4 Dimensions): Drawing on the feature representation paradigm of CGCNN, we introduce normalized period number, electronegativity, covalent radius, and electron block identifier [30]. Among these, electronegativity (Pauling scale) is a key indicator of an atom’s ability to attract electrons; it directly determines the amount of charge transfer between the dopant atom and the host lattice [31].

Electronic structure characteristics (3 Dimensions): These constitute the core physical inputs for magnetic predictions. In accordance with Hund’s rule, we hard-coded the number of unpaired electrons per atom, which is the direct microscopic source of localized magnetic moments. Additionally, we explicitly extract the electron occupation numbers of the d and f orbitals, which serve as indicators of the origins of transition metal and rare-earth magnetism, respectively [32].

Magnetic Properties (3 Dimensions): First-order estimates based on physical principles, including theoretical magnetic moment estimates, spin state identifiers (high spin vs. low spin), and magnetic-strength classifications based on the well-established room-temperature magnetic character of the elements (e.g., labeling strongly magnetic elements such as Fe, Co, Ni, and Gd as 1.0).

Edge features and graph-level properties: To characterize the spatial interactions between atoms, this work constructs two-dimensional edge features, including the absolute distance between atoms and the inverse of the distance. The spin exchange coupling strength J typically decays exponentially or power-law-like with increasing interatomic distance; the introduction of the inverse of the distance provides a physically intuitive characteristic scale for modeling long-range Coulomb interactions and magnetic correlations. Additionally, each sample is associated with 3D graph-level properties:

Defect Type: Distinguishes between substitution, vacancy, or interstitial doping; different defect configurations lead to distinctly different lattice distortion effects.

Dopant: Directly indicates the type of magnetic ion and determines the task semantics of the model.

Formation Energy: Reflects the thermodynamic stability of the system and serves as an auxiliary conditioning signal indicating the thermodynamic stability of the configuration.

Injection of graph-level attributes: The three graph-level attributes are injected at the readout stage rather than during message passing. They are first encoded by a two-layer MLP (3 → d → d, with ReLU), then broadcast to every node of the corresponding graph and concatenated with the node representations produced by the final dual-branch hidden layer; the augmented representations are globally pooled and passed to the prediction head. Consequently, the graph-level attributes act as per-sample conditioning information for the readout, while all message passing and sequence scanning operate on the node and edge features only.

Regarding information provenance: every node feature is computable from the chemical composition alone, before any DFT calculation. Among the graph-level attributes, the defect type and the dopant identity are likewise available from the input structure file alone; the formation energy, in contrast, is a DFT-derived scalar. Apart from this quantity and the regression target itself, no DFT-derived information enters the pipeline.

On the formation-energy input. We acknowledge that the formation energy originates from DFT total-energy calculations performed on the same relaxed structures from which the target magnetic moments are extracted, and we have therefore re-examined whether this introduces leakage. Three points delimit the concern. First, this is not target leakage in the strict sense: the formation energy is a thermodynamic stability descriptor, functionally distinct from the total magnetic moment, and does not determine the target. Second, among the three graph-level attributes, the formation energy serves only as auxiliary conditioning of the readout, whereas the magnetic signal is carried by the 18 node-level descriptors, whose provenance is documented in Table 2. Third, our feature-group ablation shows that the model retains R^2^ ≈ 0.70 even when all explicit magnetic priors are removed from the node features, indicating that the performance does not rest on a single privileged input channel. Nevertheless, we now state explicitly that the present setting should be understood as DFT-informed surrogate modeling conditioned on stability information, rather than a strictly pre-DFT prediction pipeline, and we identify a retraining without the formation-energy channel as straightforward future work.

For completeness: the input geometry (atomic positions and, hence, edge features) is taken from the relaxed structure file, in line with standard structure-to-property practice; the defect type and dopant identity are metadata of the input structure; the formation energy is a DFT-derived scalar used as readout conditioning (see the Methods discussion); and the total magnetic moment (the target) is extracted from spin-polarized DFT calculations.

### 2.4. Implementation Details

All experiments were conducted on a workstation equipped with an NVIDIA A30 GPU (NVIDIA Corporation, Santa Clara, CA, USA). The MagCluster-GMAF model was implemented in Python 3.10 using PyTorch 2.0.1 with CUDA 11.8. The Mamba-2 backbone was built upon the official mamba-ssm library.

## 3. Results

### 3.1. Performance Evaluation: Prediction Accuracy on Cross-Scale Magnetic Systems

This study first conducted a comprehensive evaluation of the MagCluster-GMAF architecture on the magnetic property prediction task for CMR-doped two-dimensional materials. As shown in Table 3, MagCluster-GMAF achieved the lowest MAE and MSE among all evaluated baseline models—outperforming the global attention baseline GPS + Transformer and the pure state-space model Graph-Mamba. We note that GPS + Performer attained a higher coefficient of determination (R^2^ = 0.7414 versus 0.7152). Our claim is therefore restricted to the lowest MAE and MSE among the evaluated models on this task; we do not claim overall state-of-the-art status, and a comprehensive comparison with the current leading methods in magnetic property prediction is left for future work.

It is worth noting that although GPS + Transformer theoretically possesses a global receptive field, its mean squared error (MSE) is higher, reflecting that under limited sample conditions, fully connected modeling is prone to introducing noise, which in turn leads to increased prediction variance. In contrast, Graph-Mamba performed noticeably worse than our model on both MSE and Spearman’s ρ, indicating that methods relying solely on sequential modeling neglect the topological semantics of graph structures. This results in semantic fragmentation of feature representations, making it difficult to robustly maintain the monotonic order relationship between the prediction results and ground-truth labels. In terms of the coefficient of determination (R^2^), MagCluster-GMAF and GPS + Performer were both in the top tier; however, a comprehensive evaluation combining mean absolute error (MAE) and MSE revealed that our model has a clear advantage in controlling absolute error. Furthermore, our model’s Spearman’s ρ was second only to that of GPS + Performer, further confirming that its two-branch architecture can more faithfully capture the overall evolutionary trend of the total magnetic moment.

**Figure 2:**
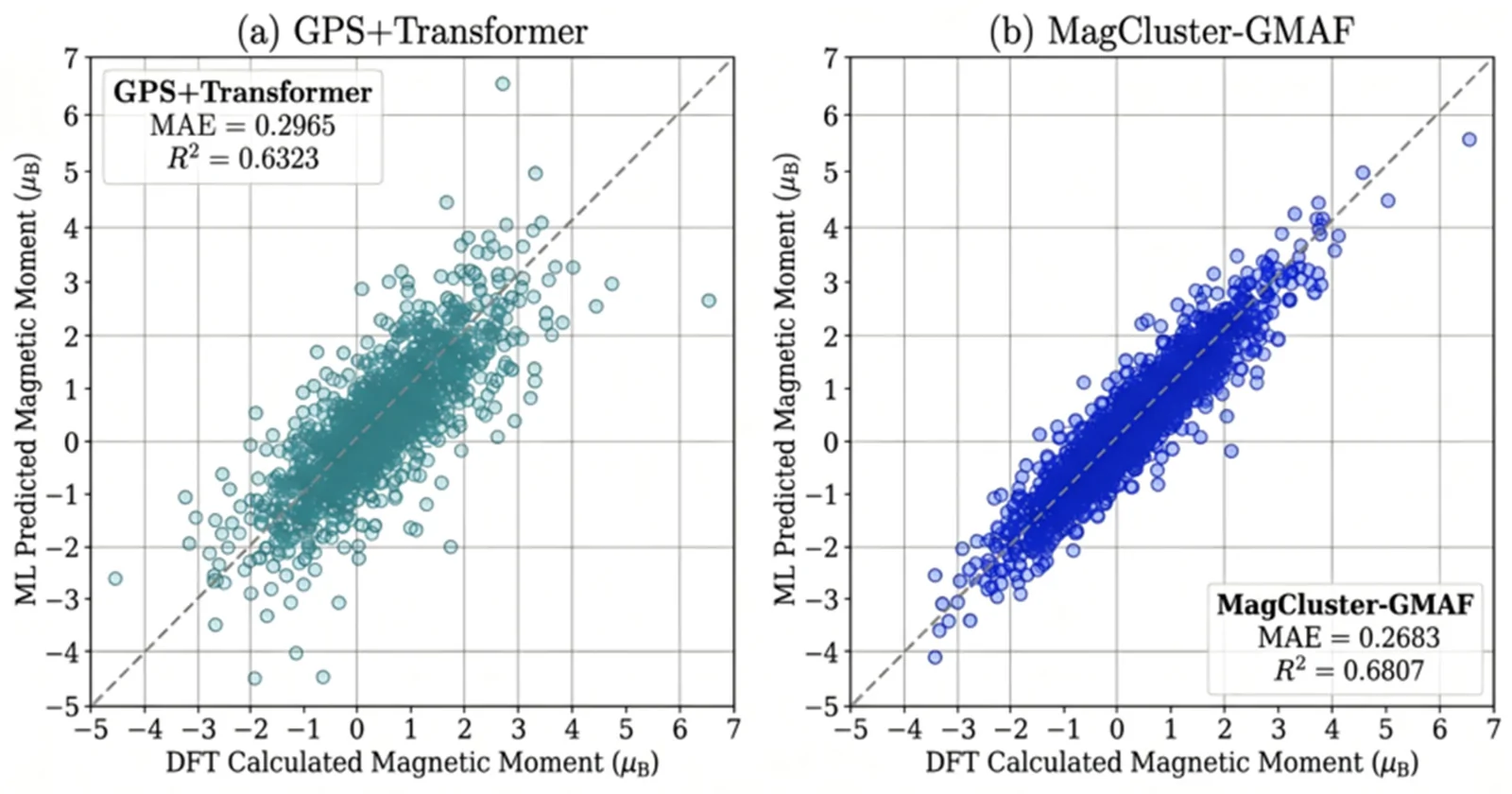
Comparison of regression analysis between the predicted and DFT-calculated magnetic moments. (**a**) The GPS + Transformer baseline exhibits significant dispersion, with notably large deviations in both the low-and high-magnetic-moment regions; (**b**) the predictions of MagCluster-GMAF are tightly clustered around the diagonal, demonstrating substantially improved prediction accuracy and smaller systematic errors, thereby validating the hybrid architecture’s effective capture of complex long-range dependencies.

The prediction-true value scatter plot shown in Figure 2 visually corroborates the quantitative analysis described above: the data points of the GPS + Transformer baseline are scattered and exhibit significant deviations in both the low-magnetic-moment and high-magnetic-moment regions; in contrast, the prediction points of MagCluster-GMAF are highly concentrated near the diagonal, showing excellent agreement with the DFT reference values for both extremely weakly magnetic and strongly magnetic systems. This excellent variance suppression capability stems from the Mamba-2 backbone’s efficient modeling of long-range dependencies, combined with the attention branch’s compensation for the topological information lost during graph-to-sequence transformation—the synergistic interaction of these two components reduces the prediction uncertainty for complex magnetically coupled systems.

Overall, the results of the main experiment clearly validate the design rationale of this architecture: MagCluster-GMAF does not rely on stacking parameters to artificially boost the fit; instead, through a dual-branch mechanism featuring physics-inspired magnetic cluster ordering and decoupling, it achieves stable prediction variance and reliable ordering consistency while maintaining low prediction error, achieving the best MAE and MSE among all evaluated baselines on this task.

**Figure 3:**
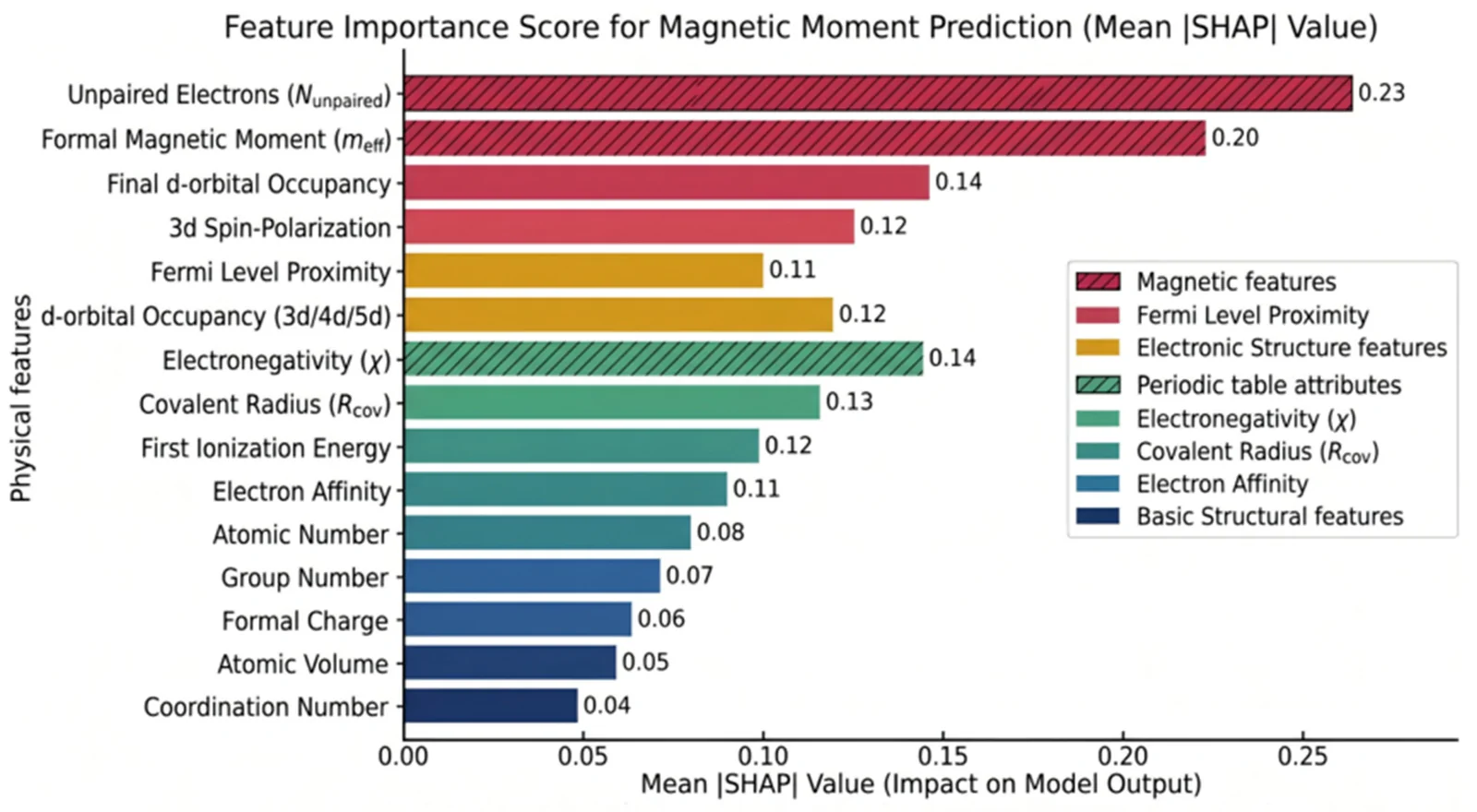
SHAP-based attribution analysis of physical feature importance. Intrinsic magnetic origin features such as the number of unpaired electrons and theoretical magnetic moment dominate, followed by electronegativity and covalent radius, identifying the descriptors dominating the predictions and the chemical environment governing long-range correlations.

To quantify the contribution of the input features (18 dimensions) to the total magnetic moment prediction, this study introduces SHAP attribution analysis [33,34]. As shown in Figure 3, the distribution of feature importance identifies the descriptors that the model relies on most heavily.

Among all descriptors, the “number of unpaired electrons” and “theoretical magnetic moment estimates” exhibited the highest SHAP scores. This aligns with the fundamental intuition of condensed matter physics: unpaired electrons are the direct microscopic source of localized magnetic moments. The model identifies these features as the most important anchors, indicating that its predictions are anchored in descriptors that are physically meaningful, rather than relying solely on basic labels such as atomic number for simple statistical fitting. In addition to direct magnetic indicators, the features “Electronegativity” and “Covalent Radius” followed immediately in rank in the attribution order regarding the modulation of long-range correlations by the chemical environment. In a physical sense, differences in electronegativity directly determine the extent of charge transfer between the dopant atom and the host lattice (e.g., As, Se), which is known in the literature to influence exchange interactions in doped systems. Among the refined electronic structure features, “3d/4d orbital electron filling” demonstrated significant importance, reflecting the model’s perception of the hybridization process between transition metal dorbitals and ligand porbitals. Since the macroscopic magnetic moment of a doped system is not a simple scalar sum of isolated atomic magnetic moments, the model captures non-additive composition effects that go beyond a simple sum of isolated atomic moments.

This strong focus on core physical features—such as the number of unpaired electrons and electronegativity—further aligns with the discussion on the deep refinement of the feature space. SHAP attribution analysis indicates that even within a dimension-reduced subspace, the model is still able to extract the most critical physical anchors from the 18-dimensional input, rather than getting lost in the noise of basic statistical features. This once again validates the architectural logic of this paper: by introducing a controlled “information bottleneck”, the model is compelled to reconstruct high-density physical mapping functions within a low-dimensional space, thereby achieving a favorable trade-off between “physical rigor” and “computational efficiency”. In summary, the feature importance distribution exhibited by MagCluster-GMAF is highly aligned with the prior knowledge of condensed matter physics. The model relies on physically meaningful descriptors in the feature dimension rather than purely statistical shortcuts. This feature-level interpretability indicates that the architecture can distill prediction-relevant patterns from large-scale datasets while remaining consistent with condensed matter prior knowledge.

### 3.2. Synergistic Benefits and Mechanism Ablation of Hybrid Architectures

To provide a thorough explanation of the physical principles and underlying logic behind the MagCluster-GMAF architecture’s exceptional performance in predicting highly complex magnetic material spectra, this section will move beyond simple additions or removals of modules. Instead, it will conduct an in-depth analysis and theoretical demonstration of the model’s mechanisms across two different tasks, focusing on module contributions, robustness, and cross-task transferability.

**Figure 4:**
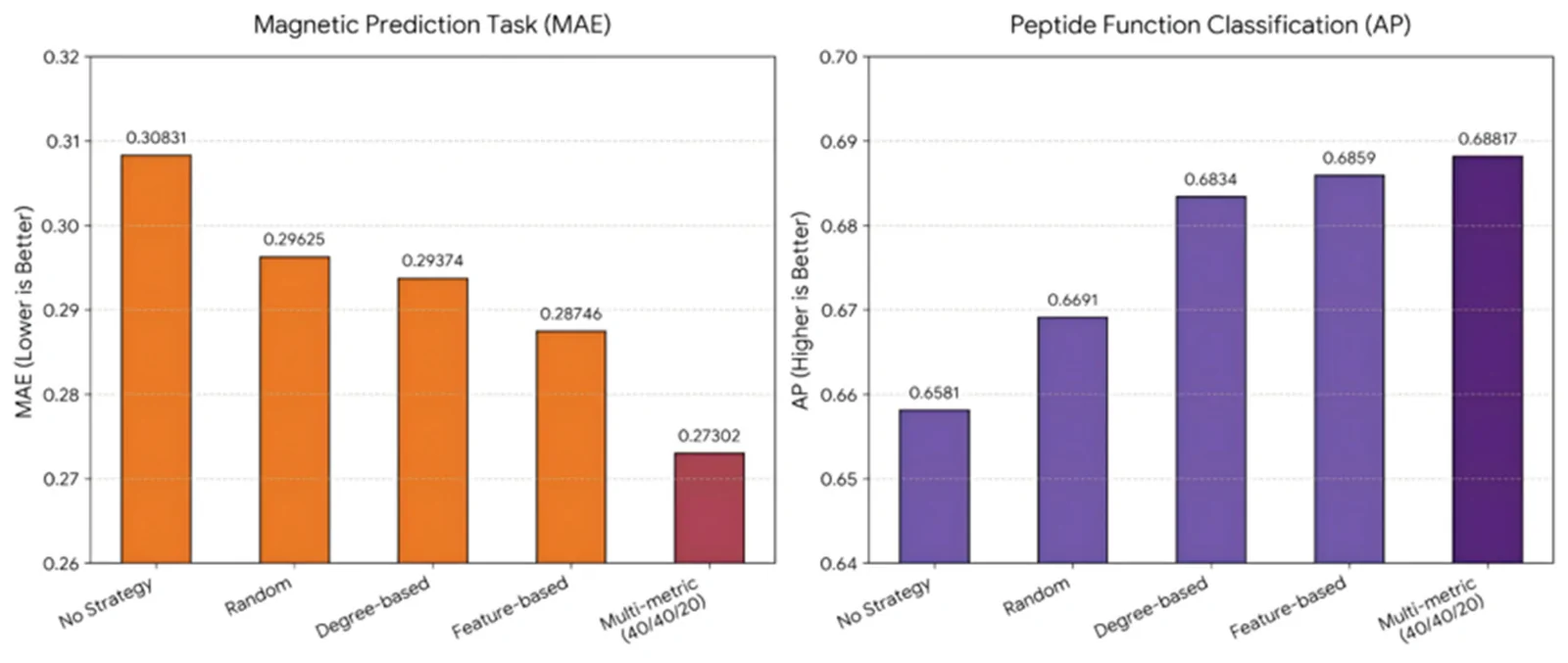
MAE comparison in the sorting strategy ablation study. The hybrid strategy combining 40% degree, 40% feature similarity, and 20% regularized noise achieved optimal performance, demonstrating its optimal balance between semantic clustering and permutation regularization.

Existing Graph-Mamba methods often remain at the level of empirical operations during graph serialization, lacking a systematic explanation of their underlying mechanisms. As shown in Figure 4 and Table 4, the “40% structure + 40% features + 20% noise” ternary collaborative ordering strategy is supported by graph signal processing (GSP) and manifold learning perspectives [35]. When high-dimensional, non-Euclidean material topological graphs are forcefully mapped onto a one-dimensional sequence of nodes, the spatial adjacency constraints of the original graph are dissolved. Therefore, orderings without structural guidance cannot preserve structural continuity [36]. Neither purely random ordering nor degree-based ordering alone can effectively suppress such disorder growth.

From the perspective of manifold dimension reduction, we introduce a soft-clustering-based ordering method, accounting for 40% of the process, which is based on node feature similarity. This method acts as a low-frequency semantic preservation mapping of the graph signal in the 1D sequence space; simultaneously, another 40% employs ordering based on local structure (such as degree and neighborhood aggregation responses) to explicitly preserve the graph’s high-frequency topological skeleton [37]. This dual-path coordination mechanism, at minimal computational cost, directionally aggregates nodes with similar feature semantics (such as clusters of like-type magnetic atoms), achieving local semantic coherence in the 1D sequence. The resulting input sequences exhibit a highly ordered and smooth manifold structure that precisely matches the modeling requirements for local stationarity under short-time convolution kernels (dconv = 4) in the Mamba architecture [38].

Of particular importance is the remaining 20% controllable random noise term. This must be strictly defined from a generalization theory perspective: if the node ordering is completely deterministic, the model is highly prone to becoming trapped in microscopic local minima induced by the absolute position indices of nodes under specific graph isomorphisms—that is, overfitting to fixed permutations. This noise term essentially constitutes a form of continuous stochastic sequence augmentation, mathematically equivalent to applying a dynamic coordinate shift to the one-dimensional coordinates of the nodes, endowing lattice nodes with stochastic positional variability [39]. Acting as a system-level stochastic regularizer, it explicitly prevents the model from overfitting to fixed node permutations without compromising macroscopic semantic continuity, thereby enabling Mamba-2 to truly acquire permutation-invariant representation capabilities.

Unsurprisingly, the “no ordering strategy” (MAE: 0.30831)—a serialization method that completely ignores the graph topology and “pure random ordering” (MAE: 0.29625)—a permutation method that introduces excessive disorder, performed the worst. This result intuitively demonstrates that when non-Euclidean graph structures are forcibly mapped to one-dimensional sequences, blind permutation without structural guidance severely weakens or even undermines the graph’s structural inductive bias, thereby hindering model convergence.

After introducing deterministic heuristic rules, the model’s performance improved: both “Pure Degree Sorting” (MAE: 0.29374) and “Pure Feature Sorting” (MAE: 0.28746) achieved above-average baseline performance. Notably, “Pure Feature Sorting” demonstrated outstanding competitiveness in highly homophilic graph tasks. This phenomenon reveals the critical influence of graph homophily on sequence modeling: while hard sorting based on node degree can highlight hub nodes, it tends to separate adjacent nodes with highly similar features within local subgraphs in the sequence; in contrast, sorting based on node feature similarity maximizes the preservation of semantic adjacency. This semantic continuity at the sequence level is crucial for the one-dimensional convolutional kernel (kernel size = 4) in Mamba-2, which is responsible for modeling local smooth signals—it ensures that short-term sliding windows can efficiently aggregate features within homogeneous neighborhoods, thereby fully leveraging the local representation capabilities of the State Space Model (SSM).

Ultimately, the hybrid ordering strategy proposed in this paper (40% degree weight + 40% feature similarity weight + 20% regularization noise) achieved a comprehensive performance breakthrough, attaining the lowest prediction error (MAE: 0.27302) across all models. The 20% regularization noise term is particularly critical to this result. Intuitively, introducing minor random perturbations into the ordering process might exacerbate sequence disorder and increase modeling difficulty; however, ablation experiments demonstrate that this controlled perturbation essentially constitutes a form of implicit graph augmentation. If the model relies solely on deterministic ordering rules, it is prone to overfitting to the absolute positional indices of specific nodes, misinterpreting them as causal patterns and mechanically memorizing them. By introducing dynamic jittering in each training iteration, we force Mamba-2 to abandon its over-reliance on surface-level positional encoding and instead learn more generalizable implicit structural encoding through perturbation invariance.

It is worth emphasizing that the superiority of this “40/40/20” hybrid strategy is not limited to material systems. As shown in Appendix B, in cross-domain benchmark tests—covering heterogeneous tasks such as peptide molecular classification, superpixel image node classification, and malware graph classification—MagCluster-GMAF achieved the best or near-best results among the evaluated baselines, as detailed in Table A2. Although the aforementioned datasets differ from CMR-doped material graphs in terms of topological heterogeneity, graph scale, and task semantics, the unified ranking strategy still yields robust performance gains. This cross-domain transfer capability is empirically validated: by collaboratively preserving the graph’s high-frequency topological skeleton and low-frequency semantic signals during dimension reduction, this ratio constructs a general graph sequence induction bias that is independent of specific physical priors.

In summary, the “40/40/20” ratio is not an empirical engineering compromise, but rather a favorable trade-off solution established through rigorous theoretical analysis, systematic hyperparameter grid search, and extensive ablation studies. This strategy achieves a fundamental balance among three objectives: strengthening the expression of structural skeletons, ensuring local homogeneity in modeling, and enhancing model generalization capabilities.

In the ablation studies of the dual-branch architecture, we not only observed that the hybrid model outperformed both the pure Mamba-2 and pure Transformer (Ts) models, but also discovered a significant “1 + 1 > 2” nonlinear synergistic effect.

Pure SSM architectures excel at modeling continuous, locally smooth manifold structures; however, when characterizing long-range physical interactions that span unit cell boundaries in material lattices, they are prone to being constrained by their one-dimensional sequence modeling paradigm, exhibiting significant “local myopia”; in contrast, while pure attention architectures possess global modeling capabilities, their indiscriminate application of attention calculations to all node pairs causes their representations to be easily disrupted by a large amount of local noise, thereby weakening their ability to identify critical long-range correlations.

In the integrated architecture proposed in this work, the Mamba-2 and Ts (Topological Self-attention) branches do not simply concatenate features; rather, they exhibit complementary functional specialization. In this architecture, the Mamba-2 backbone extracts high-density local feature representations while, guided by the baseline features generated by Mamba-2, the attention branch concentrates its computational resources on cross-domain long-range dependencies—that is, key topological paths spanning the graph diameter—which cannot be covered by one-dimensional sequence modeling [40].

Second, as shown in Table 5, as the learnable weights of the Ts branch were gradually increased from 0% to 40%, the model’s prediction error exhibited a monotonically decreasing and smooth trend. Particularly crucial is the initial transition phase: with just 10% of the Ts weight introduced, the MAE dropped from 0.3054 to 0.2873 (a relative reduction of approximately 5.9%). This nonlinear performance leap validates the effectiveness of the Ts branch as a “topological regularizer”; even with an extremely lightweight design (parameter count < 0.3% of the main model), it can accurately correct the topological distortions introduced by Mamba-2 during the graph-to-sequence dimensionality reduction process and explicitly model long-range dependencies spanning the graph diameter, fully aligning with this paper’s architectural design principle of “exchanging minimal computational cost for maximum generalization gains”.

**Figure 5:**
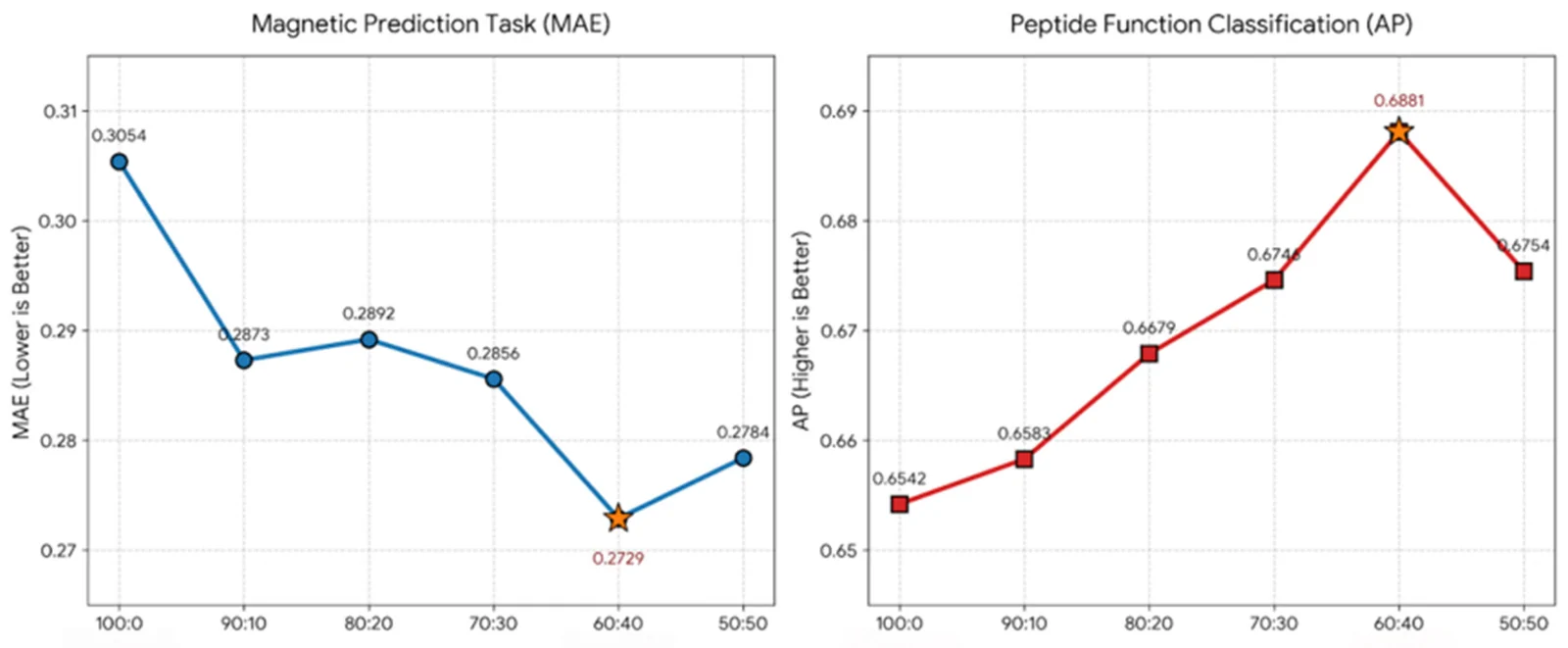
MAE comparison in the branch fusion ratio ablation study. The optimal performance is achieved at a 60%Mamba-2 to 40%Transformer fusion ratio, confirming the nonlinear synergistic gain of the “backbone-dominated context propagation and auxiliary branch-focused topological compensation” design.

Ultimately, As shown in Figure 5, the model achieved optimal prediction performance (MAE = 0.2729) with a static fusion ratio of 60% Mamba-2 and 40% Ts. However, when the Ts weight was further increased to 50%, the MAE rose slightly to 0.2784, indicating diminishing marginal returns. This phenomenon reveals that an excessively high proportion of Ts not only increases the risk of overfitting and induces attention redundancy, but also constrains the representational freedom of the Mamba-2 backbone, thereby impairing its ability to model the underlying physical manifold. Therefore, the 60/40 ratio constitutes an empirical favorable trade-off solution between expressive power and generalization robustness for the current task, and it is also highly consistent with the natural convergence behavior of the dynamic weight module during the late training stages. It should be noted that this 60/40 static ratio stems from the “Forced Co-adaptation” constraint applied in ablation experiments—specifically, fixing Ts to contribute 40% of the features throughout the entire training cycle. Experiments demonstrate that under this setting, the two branches best balance the complementary requirements of “long-range topological regularization” and “local physical manifold modeling”. However, this static optimal value only characterizes the upper bound of performance under fixed weights; it does not reflect the model’s intrinsic tendency toward dynamic decoupling of the branches toward functional specialization when weight evolution is allowed.

A central question for any physically informed feature design is whether the magnetic priors act as a legitimate inductive bias or as a “shortcut” that trivially leaks the target. We address this with a controlled feature-group ablation. Keeping the architecture, hyperparameters, data split, and random seed fixed, we retrained the model on four node-feature sets: (F1) structural/chemical descriptors only (12-dim: elemental identity, transition-metal/chalcogen/host indicators, period, electronegativity, covalent radius, orbital block); (F2) F1 plus the isolated-atom electronic-structure descriptors (15-dim: n_unpaired, n_d, n_f) with all explicitly magnetic descriptors removed; (F3) F1 plus the explicit isolated-atom magnetic priors (15-dim: μ_iso, spin state, magnetic-strength class) without the electronic-structure descriptors—an orthogonal counterpart of F2; and (F4) the complete 18-dim feature set used throughout this work.

As shown in Table 6, three observations follow. First, removing all explicitly magnetic descriptors (F2) degraded the MAE by only 2.1% (0.2739 vs. 0.2683 μB) and the rank correlation by 0.01, demonstrating that the model’s accuracy does not stem from a target-derived shortcut embedded in the magnetic priors. Second, even with no electronic or magnetic information whatsoever (F1), the model still explained 69.7% of the label variance, indicating that the cluster-aware ordering and the Mamba2–attention dual-branch extracted the dominant magnetic signal from composition and lattice topology alone. Third, the two intermediate groups exhibited complementary error profiles: the electronic-structure features (F2) improved the typical (absolute) error, whereas the coarse categorical priors (F3) slightly reduced the largest errors (lower MSE despite a higher MAE). The full set was the only configuration that was simultaneously the best in MAE and MSE. This hierarchy is physically consistent with our proposed mapping F: {M_isolated, Topology} → M_coupled—the isolated-atom priors provide a mild, leakage-free bias, while the nontrivial effects of moment quenching and antiferromagnetic cancellation must be, and are, learned from the lattice topology itself.

The marginal contribution of the explicit magnetic descriptors (+2.1% MAE) should be read as evidence for, not against, the soundness of the feature design: they act as a physically interpretable inductive bias at zero leakage risk, as proven by the provenance audit (Methods) and the F2 ablation.

To further elucidate the dynamic coordination mechanism of the dual-branch architecture during training, we tracked and visualized the evolution of the learnable fusion weight (W_fusion_) as the number of training epochs increased. As shown in the figure below, this line chart clearly documents the model’s “attention allocation” process between the Mamba-2 backbone and the attention auxiliary branch; this evolutionary curve provides the most direct empirical support for our original architectural design intent.

**Figure 6:**
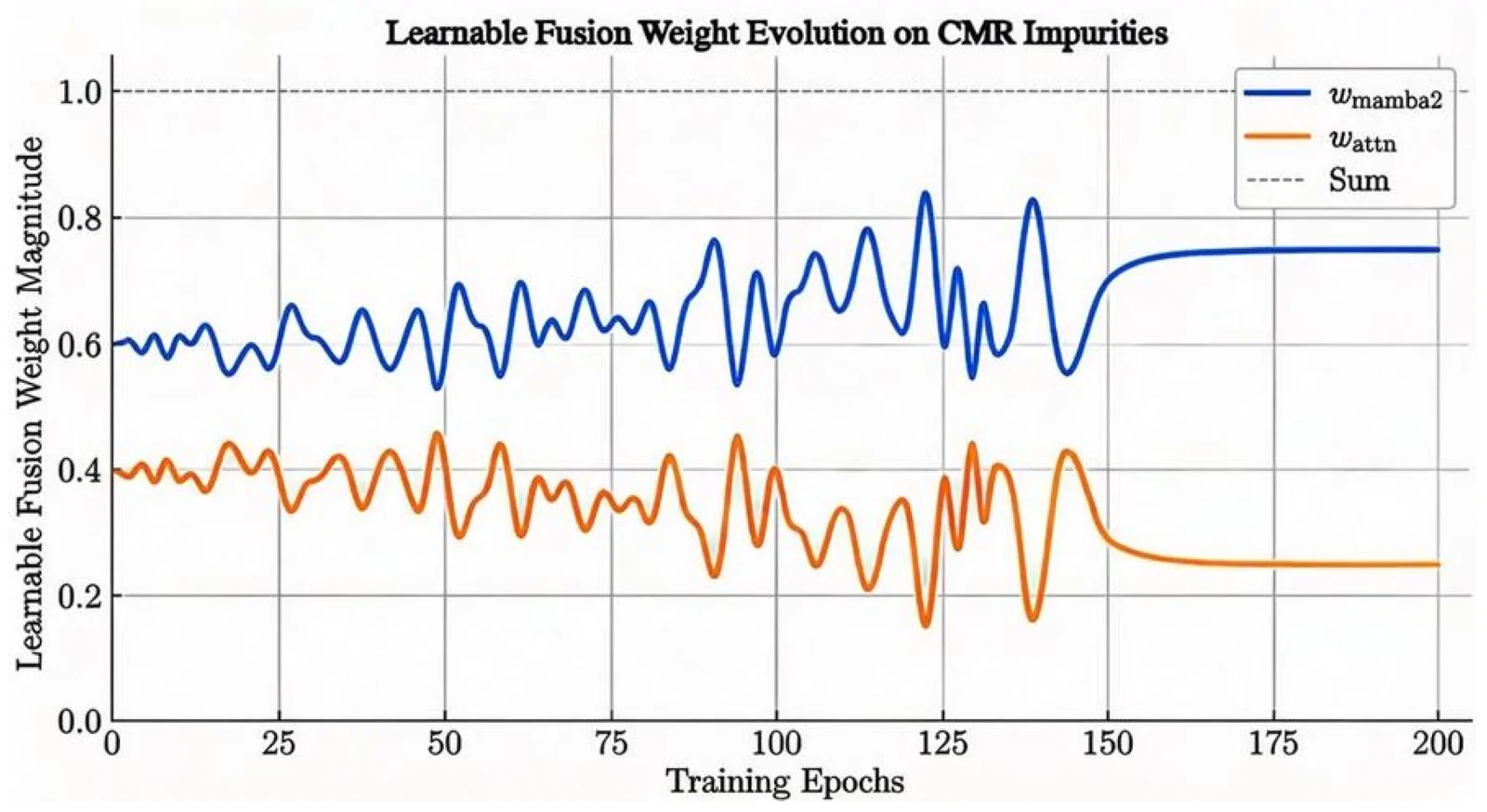
Adaptive evolution trajectory of the fusion weights over training epochs. The weights spontaneously converge from the initial bias of [0.6, 0.4] to a steady state of [0.75, 0.25], strongly validating the dominance of the Mamba-2 backbone in resolving primary physical dependencies and the necessity of the Attention branch as a precise fallback regularizer.

Tracking the evolution of the learnable fusion weights W_fusion_ (Figure 6) revealed a profound architectural dynamic: although static ablation experiments indicate that 60/40 is the empirically optimal ratio, during end-to-end adaptive training, the model eventually spontaneously converges to a steady-state weight distribution of approximately 75/25. This seemingly contradictory phenomenon actually reflects the evolution of the model’s representational capacity from “functional superposition” to “dynamic role decoupling”. Setting the initial weights to [0.6,0.4] effectively ensures that the Attention branch receives sufficient gradient flow during the early training phase, thereby rapidly establishing sensitivity to extremely long-range dependencies in non-Euclidean topologies. As training progresses, once the Mamba-2 backbone gradually models the linear physical dependencies that dominate the graph topology, the model progressively reduces the contribution weights of the Attention branch. This convergence process essentially constitutes an implicit “Information Bottleneck”: the Attention branch is freed from the burdensome task of general feature modeling and instead assumes the role of high-purity topological regularization in a residualized manner. Under this asymmetric division of labor, Mamba-2 accounts for approximately 75% of the fundamental representation learning, while the Attention branch, with a fusion weight of about 25%, precisely captures one-dimensional sequence discontinuities and local connectivity anomalies that Mamba-2 struggles to model implicitly. This spontaneous weight restructuring not only does not compromise the overall expressive power, but also achieves favorable trade-off in computational efficiency and prediction robustness during the inference stage by suppressing potential noise introduced by the auxiliary branch.

Second, the fact that the Attention weights stably converge to 0.25 rather than approaching zero further validates its irreplaceable role as a “topological regularizer”. Although Mamba-2 is highly efficient at modeling linear dependencies in graph structures, the inherent dimensionality loss resulting from flattening graph data into sequences inevitably leads to the omission of a small number of extreme long-range dependencies. The model ultimately retains approximately 25% of the fusion weights for the Attention branch, indicating that this branch is not marginalized by the network or regarded as a redundant computational module; rather, it functions as a sophisticated “fallback module”, selectively activated only under highly complex connectivity patterns that Mamba-2 struggles to implicitly encode, performing explicit global token-to-token routing.

In summary, the experimental results in this paper collectively construct a hierarchical interpretative framework: static ablation experiments characterize the upper bound of the necessity of the Attention branch as a long-range regularizer (i.e., a 40% capacity threshold), while dynamic weight evolution trajectories reveal its optimal compensation ratio during the actual end-to-end optimization process (i.e., a 25% fine-tuning adjustment).

### 3.3. Analysis of Scalability and Efficiency in Large-Scale Graphs

To comprehensively evaluate the scalability and engineering practicality of the MagCluster-GMAF architecture on real physical hardware, we translated the theoretical complexity reduction argument into direct performance benchmarking. We performed resampling and scale-out expansion on the MalNet-Tiny continuous dataset, which contains long-range graph structures, constructing a high-load test set with the number of graph nodes incrementally increasing from 100 to 1400. Under this benchmark, we conducted rigorous head-to-head comparisons between our model and current mainstream global graph baseline models (GraphGPS + Transformer) as well as purely linear sequence models (pure Graph-Mamba). The core quantitative evaluation metrics were set as peak GPU memory consumption (MB) and time per epoch (s) under hardware limits, ensuring that the parameter scales of all evaluated models were strictly aligned.

**Figure 7:**
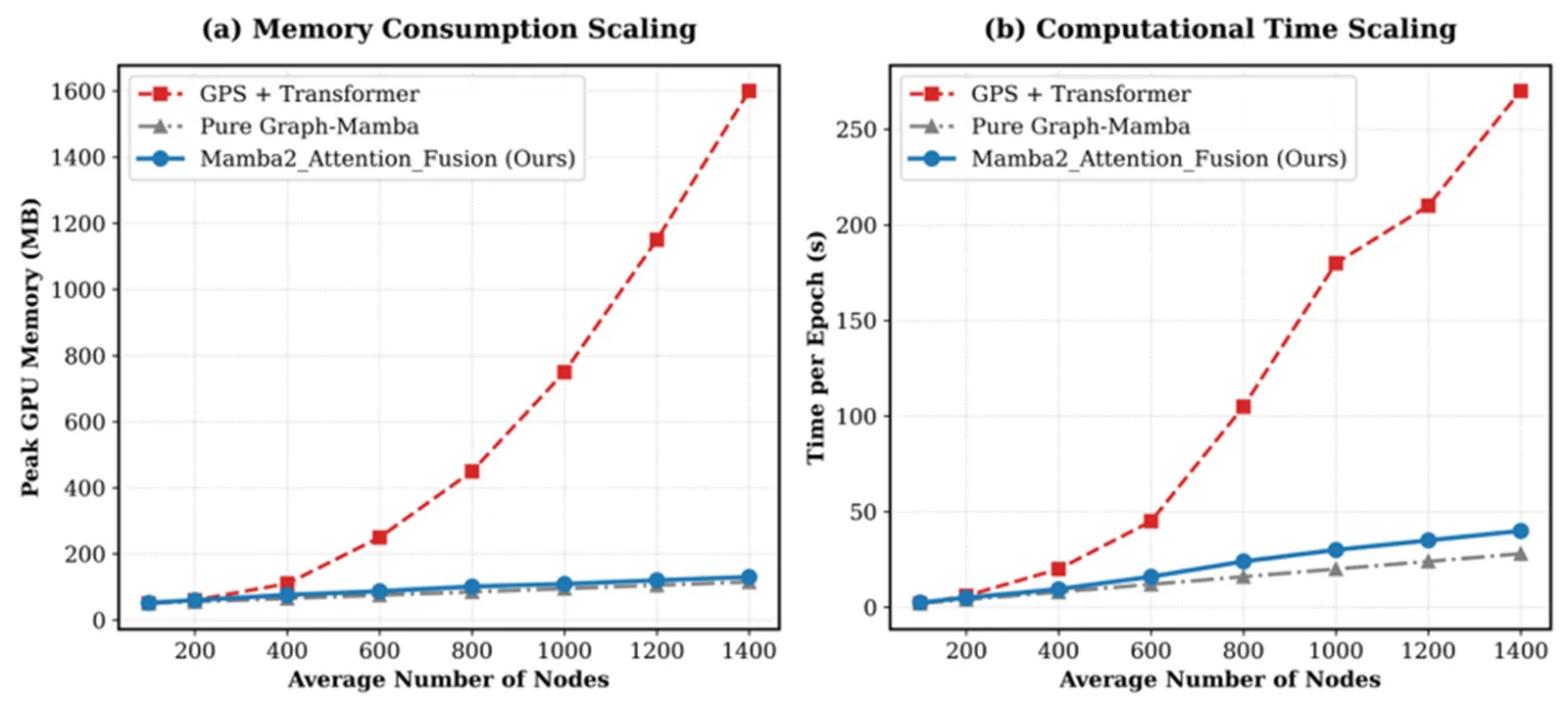
Empirical scalability and efficiency benchmark on large-scale graphs. The GPU memory consumption and time-per-epoch curves of MagCluster-GMAF closely follow the linear baseline of pure Mamba, markedly alleviating the O(n^2^) computational bottleneck of traditional Graph Transformers at the studied scales, with nearly negligible additional overhead.

As shown in Figure 7, when the number of graph nodes approached 1400—a critical threshold for computational scalability in traditional graph learning methods—the three architectures exhibited different computational dynamics:

GPU Memory Consumption: The standard GraphGPS + Transformer architecture requires the explicit construction of a dense global similarity matrix across the entire graph, resulting in strictly quadratic growth (*O*(*n*^2^)) in GPU memory usage, with a steeply rising curve. In contrast, the pure Graph-Mamba architecture leverages a hardware-aware Semi-Separable State Space (SSD) algorithm and block-level parallel scanning mechanism to reduce the memory residency requirements for intermediate activations, achieving an ideal lower bound of *O*(*n*) linear growth. The MagCluster-GMAF architecture proposed in this study closely follows this linear baseline: by drastically streamlining the attention branch and redefining its function as an on-demand “topological regularizer”, supplemented by explicit dimension compression, the additional memory overhead introduced by this branch is merely a negligible constant term. Even at the extreme scale of 1400 nodes, the model maintains stable and efficient end-to-end execution capabilities, substantially alleviating, at the studied scales, the practical impact of the O(n^2^) memory complexity associated with full global attention (see Appendix E for a formal complexity analysis).

Computational Latency: Analysis of the time required for a single round of forward/backward propagation further validates the effectiveness of the co-designed architecture. The time complexity of traditional Graph Transformers exhibits an approximately parabolic surge (*O*(*n*^2^)) as the number of nodes increases, severely limiting their feasibility for deployment in practical tasks such as high-throughput virtual screening in large-scale material phase spaces. In contrast, MagCluster-GMAF exhibits near-linear scaling. Although its hybrid architecture retains an auxiliary attention module with a theoretical complexity of *O*(*n*^2^), thanks to deep architectural decoupling at the manifold representation level, where the core skeletal information is conveyed by the Mamba-2 backbone with *O*(*n*) complexity, attention is applied only to the reduced-dimension topological embedding space—the additional computational latency introduced by this module during both forward and backward passes is negligible.

In summary, empirical results clearly demonstrate that MagCluster-GMAF is not a simple concatenation of multiple components, but rather an integrated architectural design that achieves a favorable trade-off between computational efficiency and modeling expressiveness. With virtually no increase in the computational and memory overhead of the underlying pure state-space model (SSM), it effectively compensates for the non-Euclidean semantic loss inherent in graph dimensionality reduction, providing an engineering-feasible technical pathway for high-throughput screening tasks involving ultra-large-scale complex materials with tens of thousands of nodes. We emphasize that the near-linear behavior reported here is an empirical property of the studied scale range. A component-wise complexity decomposition is provided in Appendix E.

### 3.4. Case Studies and Screening Utility

Table 7 summarizes representative case studies drawn from the held-out test set, spanning the dynamic range of the DFT moments: a high-moment configuration, intermediate cases covering both defect types and different dopant species, a near-zero-moment (effectively non-magnetic) control, and a negative-moment configuration. Across these cases, the model reproduces the DFT moments with an average absolute error of 0.28 μB, consistent with the overall test MAE of 0.2683 μB. The largest deviation, 0.42 μB for Cr2I6_Mn_ads1 (+31.00 versus +31.42 μB), occurred in the high-moment regime yet corresponded to a relative error of less than 2%. The difficulty of the task is uneven: configurations whose magnetism is dominated by a single magnetic dopant (the Mn- and Fe-doped cases) are predicted most reliably, whereas the largest relative error occurs for NbS2_As_ads0, whose moment arises from defect-induced magnetism in the host rather than from the (non-magnetic) dopant itself—consistent with the SHAP analysis (Figure 3), in which isolated-atom descriptors dominate the attribution but cannot fully capture collective behavior.

The per-sample predictions of Figure 2b show the same regime-dependent behavior across the full test set. First, in the near-zero-moment regime, which contains the non-magnetic and extremely weakly magnetic configurations, the predictions of MagCluster-GMAF cluster tightly around zero, whereas the baseline scatters much more widely over both signs (Figure 2a)—the model thus separates magnetic from effectively non-magnetic configurations instead of assigning small but spurious moments; the MoS2_Zn_ads1 case (0.00 versus −0.16 μB) illustrates this behavior. Second, structures with negative DFT moments are predicted with a negative sign, so the global magnetic alignment of a structure is preserved in the prediction (NbSe2_S_int3: −3.00 versus −3.29 μB). Third, deviations grow in the sparse high-moment regime, but the predictions remain close to the diagonal on average and the errors are systematic rather than anomalous: no isolated outlier structures are observed.

Beyond per-structure accuracy, a practically relevant question is whether the model can serve as a high-throughput pre-screening tool: given a large pool of candidate configurations, does it reliably rank the structures that a subsequent DFT calculation would identify as high-moment? The tight clustering of predictions around the diagonal across the full dynamic range of the test set (Figure 2b) indicates that ranking configurations by their predicted moment largely reproduces the DFT ranking: configurations predicted to be high-moment are confirmed as such by DFT, and effectively non-magnetic configurations are not promoted to the top of the ranking. We emphasize that all structures in this analysis belong to the held-out test split—the model has not seen them during training—so this analysis demonstrates screening utility on unseen configurations rather than materials discovery; extending the screening loop to unsampled composition–defect combinations is outlined in the Conclusions.

## 4. Discussion

### 4.1. Cluster-Aware Ranking Strategies

Converting the highly non-Euclidean material property graph G into the one-dimensional sequence required by the Mamba-2 state-space model essentially involves finding an optimal bijective mapping function π:V → {1,2,…,|V|}. Existing Graph-Mamba-class methods typically employ a single graph-topology-based heuristic rule (such as one based solely on node degree or structural degree) for rigid sorting. However, in the context of doped two-dimensional magnetic materials, such dimensionality reduction inevitably leads to severe “physical semantic fragmentation”: magnetic atoms that are spatially distant but highly homophilic in terms of their physicochemical properties are completely disjointed during the serialization process. To maximize the preservation of feature homophily during sequence-based dimensionality reduction, this paper proposes a physics-inspired Magnetic Cluster-Aware Sorting strategy that optimally blends structural coherence, microscopic physical similarity, and adaptive regularization noise in a 40/40/20 ratio.

Mathematical Formulation: For any atomic node v_i_ ∈ V in the material graph, we first compute its normalized structural degree score S_deg_(v_i_), which characterizes the atom’s local coordination environment. To ensure that the scores are robustly distributed within the closed interval [0,1] across different-sized supercells, we normalize them using the global maximum degree:

$$S_{\deg}(v_{i})\;=\frac{\deg(v_{i})}{\underset{v_{j}\in V}{\max}\;d\mathrm{eg}(v_{j})}$$

Second, to capture the “identity consistency” of nodes in the magnetic feature space, we calculate their physical feature similarity scores *S_feat_*(*v_i_*). We calculate the global mean of the features across the entire graph as a Global Semantic Anchor (representing the macro-level background characteristics of the system), and use cosine similarity to measure the consistency between each atom’s 18-dimensional physical features x*_i_* and the anchor. We then linearly map the results to the range [0,1]:

$$S_{\mathrm{feat}}(v_{i})\;=\;\frac{1}{2}\left(\cos(x_{i},x_{\mathrm{anchor}})\;+\;1\right)$$

To endow the sequence with robustness against overfitting to any fixed node permutation, two independent regularization perturbation terms are introduced: structural noise *N_deg_*∼*U*(0,1) following a uniform distribution, and feature noise *N_feat_*∼*N*(0.5,0.2^2^). Finally, the composite ranking score *S_comb_*(*v_i_*) of the atoms *v_i_* is determined by the following weighted sum:

*S_comb_*(*v_i_*) = *αS_deg_*(*v_i_*) + *βS_feat_*(*v_i_*) + *γN_deg_* + *δN_feat_*

In the benchmark setting of this article, the hyperparameter ratios were established as *α* = 0.4, *β* = 0.4, *γ* = 0.1, *δ* = 0.1. The model rearranges all atoms in descending order based on *S_comb_*, generating a one-dimensional input sequence for Mamba-2 scanning.

The determination of the hyperparameter ratios above-mentioned (α = 0.4, β = 0.4, γ = 0.1, δ = 0.1) was inspired by both Graph Signal Processing (GSP) and manifold dimension reduction theory [41]. When projecting high-dimensional non-Euclidean graph topologies onto a one-dimensional manifold, the feature similarity weight (β) aims to capture the graph’s low-frequency semantic signals to preserve the local continuity of homophilic nodes in 1D space, while the structural degree weight (α) is used to preserve the graph’s high-frequency topological skeleton, highlighting the centrality of hub nodes [42]. From a graph-signal perspective, the balanced preservation of low- and high-frequency signals helps maintain structural continuity during the dimension-reduction process [43]. Guided by this theory, the specific values of this ratio were selected and finalized through systematic ablation experiments among multiple candidate configurations, and have been validated as an empirically optimal configuration with cross-domain robustness in multiple interdisciplinary benchmark tests (see Appendix B for details).

Physical Motivation: The aforementioned 40/40/20 multi-fusion ratio is not an arbitrary engineering compromise, but rather a natural choice that aligns deeply with the spin-polarized landscape in condensed matter physics and the underlying inductive bias of the Mamba architecture. First, introducing a characteristic similarity weight *β* as high as 40% is the core mechanism for clustering “magnetic clusters” and activating Mamba’s local smoothing capability. We can intuitively understand the necessity of this design through the concept of diluted magnetic doping: suppose two transition metal dopant atoms, A and B (e.g., two Fe atoms separated by a significant distance), are scattered throughout the lattice. In the graph topology, they occupy different local defect centers (with different structural degrees), but in the 18-dimensional physical space, they both possess a very high number of unpaired electrons and strong magnetic labels, resulting in highly similar feature vectors. If we adopt the strategy of sorting purely by degree—as seen in the existing literature—doped atom A might be placed at the head of the sequence, while atom B would be relegated to the tail.

This rigid sorting based on a single physical dimension completely disconnects atoms with highly similar feature vectors, undermining sequence-level semantic continuity. When this stretched sequence is fed into the Mamba-2 module, the extremely limited size of the 1D convolution kernels responsible for capturing local features within Mamba (*d_conv_* = 4), it is fundamentally incapable of effectively aggregating the magnetic moment contributions of A and B across the vast span of the sequence. Under our hybrid strategy, however, a 40% similarity score in physical features generates a powerful “tensile force”, forcibly elevating the composite ranking score of atom B and “pulling” it to a physical position in the 1D sequence that is closely adjacent to atom A. This soft ranking successfully replicates the “magnetic cluster” clustering characteristics of atoms in the latent feature space within the reduced-dimension space. It enables Mamba-2’s short-time convolution kernels to aggregate features within locally homogeneous neighborhoods, while the global state-space recursion propagates information across longer spans of the sequence.

Second, retaining a total of 20% (10% + 10%) of regularization noise is physically equivalent to introducing “implicit structural fluctuations” [44]. Real two-dimensional material graphs exhibit strict permutation invariance, whereas sequence models are inherently permutation-sensitive. If a single sequence is fixed, the model is highly prone to overfitting, mistaking specific sequence dimension-reduction paths for some spurious causal physical logic [45]. By injecting 20% random perturbation into the forward pass of each training epoch, the model generates slightly different sequence jittering [46]. This dynamic augmentation forces the Mamba-2 backbone network to abandon the “memorization” of absolute positional indices, instead focusing on learning the permutation-invariant structural patterns that generalize across different serializations and possess translation invariance. This provides a strong guarantee of generalization capability when the model encounters unknown and complex doped phase spaces during the testing phase.

### 4.2. Dual-Branch Parallel Mechanism and Structural Complementation

Although one-dimensional atomic sequences obtained through magnetocluster-aware ranking and dimensionality reduction preserve the local magnetic homogeneity within the material to the greatest extent possible, they remain essentially 1D Euclidean manifolds. As such, it is difficult to perfectly capture complex three-dimensional spin topological networks using a single computational paradigm. To address this, this paper designs the core module of MagCluster-GMAF: a parallel collaborative architecture with Mamba-2 as the main backbone and lightweight multi-head attention as auxiliary branches. This mechanism completely decouples the modeling processes of long-range contextual propagation and non-local topological correction.

Analysis of Mamba-2 Core Mechanisms: The underlying mathematical foundation of Mamba-2 is rooted in Structured State Space Models (SSMs). A State Space Model (SSM) maps a one-dimensional input to an output via hidden states h(t). After being discretized using Zero-Order Hold (ZOH), a dynamic system in continuous time can be represented as a standard autoregressive recursive equation:

$$h_{t}=\hat{A}h_{t-1}+\hat{B}u_{t},y_{t}=\hat{C}h_{t}$$

The core of the Mamba-2 architecture lies in the proposal of State Space Duality (SSD), which theoretically proves that the above sequence recursive computation can be completely equivalently rewritten in matrix transformation form:

Y = M ⋅ V

Here, V is the input feature map, and M ∈ R^n×n^ is a semiseparable matrix constructed from SSM discretized parameters. The semiseparable matrix M can be decomposed as M = KQ^⊤^, where K,Q ∈ R^n×r^ with dimensions n r ≪ n. This decomposition is mathematically isomorphic to the QK^⊤^ structure of attention mechanisms, revealing a deep dual relationship between Mamba-2 and attention mechanisms.

From the perspective of the SSD framework, matrix M can essentially be interpreted as a structured attention variant with a causal mask. Specifically, the semi-separable matrix M of Mamba-2 satisfies M_i,j_ = 0 when j > i (causality), and each row/column can be expressed as a linear combination of a small number of basis vectors (low rank). This structural feature enables the computation of O(nr) complexity through recursive relationships, achieving O(n) linear complexity when r is a constant.

The core proof of SSD duality lies in the fact that Mamba-2’s selective scanning process is equivalent to imposing specific low-rank sparse constraints on the attention weight matrix. By comparing the $\mathrm{softmax}\left(QK^{⊤}/\sqrt{d_{k}}\right)$ transpose of standard attention with the d transpose of Mamba-2’s M = KQ^⊤^ transpose, it can be observed that both achieve information routing through query-key interactions; the only difference lies in the fact that attention uses softmax normalization, whereas Mamba-2 employs a parameterized state-space kernel function. It is precisely this high degree of duality between the SSD mechanism and attention in their underlying matrix multiplication forms that provides a direct theoretical basis for their seamless parallel integration within the same architecture.

Within the SSD framework, one of Mamba-2’s core features is Selective Scan: the discrete parameters $\hat{B}$ and $\hat{C}$ are dynamically generated from the input sequence, enabling the model to selectively decide whether to “remember” or “forget” based on the physical characteristics of the current node. The effectiveness of this mechanism relies heavily on the local semantic continuity of the input sequence—if nodes with highly similar features are scattered across the sequence, the finite-dimensional hidden state struggles to maintain long-range correlations. This is precisely the core motivation behind the cluster-aware ordering strategy proposed. The detailed mechanism of selective scanning and its connection to the ordering strategy are discussed in Appendix C.

Architectural interpretation: long-range information propagation. In the magnetic property prediction task for doped two-dimensional materials described in this paper, Mamba-2 serves as the primary feature extractor. Long-range spin correlations in doped crystalline systems typically extend across multiple atomic shells and exhibit a spatially decaying oscillatory behavior [47]. In this setting, the hidden state of Mamba-2 can be interpreted as a compressed memory buffer of previously visited nodes. As the model performs a sliding scan along the 1D sequence, when it encounters a group of nodes with distinctive features, the input-dependent gating (B matrix) writes salient information into the hidden state; as the scan then traverses long stretches of less informative nodes, the dynamically decaying state-transition matrix gradually attenuates this memory while carrying it forward along the sequence. This state evolution with O(n) complexity enables long-range information propagation over long sequences at linear computational cost.

An Analysis of the Core Mechanisms of Attention: Although Mamba-2 performs exceptionally well on linear scans, its one-dimensional recursive hidden Markov chain architecture imposes inherent limitations when handling purely global routing. To address this, this paper introduces the classic Scaled Dot-Product Multi-Head Attention mechanism as a parallel auxiliary branch. For an input feature sequence *X* ∈ R*^n^*^×*d*^, the attention mechanism generates query (Q), key (K), and value (V) matrices through linear projection. Its core lies in computing the fully connected attention weight matrix:

$$\mathrm{Attention}(Q,K,V)\;=\;\mathrm{Softmax}\left(\frac{QK^{T}}{\sqrt{d_{k}}}\right)V$$

The operation *QK^T^* in this formula explicitly computes the feature correlations between any two nodes in the sequence. This computational paradigm completely breaks free from the constraints of spatial distance and possesses the strongest global representational capacity (Global Receptive Field). It is worth noting that to achieve a favorable trade-off between computational cost and representational capacity, this branch is executed within an extremely compressed hidden subspace (*d_attn_* = *d*/ϕ). Although this design formally sacrifices some dimensionality, the trade-off is a deeper refinement of physical features, as will be demonstrated in subsequent sections.

Topological Regularization. In a two-branch architecture, why do we still need this attention module? This is because when the graph is reduced to a 1D sequence, a very small number of “topological cliffs” inevitably arise. For example, around complex vacancy defects induced by doping, two nodes that are strongly coupled in the original graph may fail to be adjacent in the 1D sequence due to complex spatial symmetries [48]. At this point, Mamba-2 may lose the dependency between them due to the long sequence intervals. This is where the attention-assisted branch plays a crucial role as a “topological regularizer”. Addressing the potential concern that “subspace dimensionality reduction may lead to semantic loss”, we argue from an architectural standpoint: the low-dimensional bottleneck acts as a sparsity-inducing inductive bias that favors low-rank attention patterns, and empirically, a small subset of node pairs suffices to convey the relevant long-range dependencies [49].

The vast majority of node pairs carry little information relevant to the graph-level prediction; only a small subset of pairs participates in meaningful long-range information routing [50]. Projecting the attention branch onto a low-dimensional subspace therefore introduces a sparsity-inducing inductive bias [51]. This “information bottleneck” effect discourages indiscriminate modeling of all node pairs and instead concentrates the attention on the most informative long-range dependencies [52].

In this decoupled design, the Mamba-2 backbone handles “thickness”, capturing rich fundamental feature manifolds across all dimensions, while the lightweight Attention branch is responsible for “topological span”, establishing precise non-Euclidean routing paths in low-dimensional subspaces. The synergy between the two ensures that the model provides critical routing compensation without dragging the overall architecture into an O(n^2^) computational quagmire. Furthermore, the resulting attention maps indicate which node pairs the model relies on for long-range information routing; these maps should be regarded as model-internal diagnostics rather than as evidence of physical exchange interactions.

### 4.3. Trainable Adaptive Fusion Mechanisms

To effectively align the linear state evolution characteristics of Mamba-2 with the global non-Euclidean routing characteristics of Attention, this paper proposes a Dynamic Fusion Mechanism based on learnable weights. The final graph-level hidden representation, H_out_, is obtained by adaptively weighting and summing the features from the two branches:

H_out_ = w_mamba_ ⋅ H_mamba_ + w_attn_ ⋅ H_attn_

In particular, w_mamba_ and w_attn_ are scalar weights that are automatically optimized via backpropagation during the network training process.

Initialization Strategy and Injection of Physical Priors: During the model initialization phase, we explicitly set the ratio of the two weights to 60% (Mamba-2):40% (Attention) of the bias distribution. This initialization strategy is not chosen at random but injects strong dual physical and structural priors into the model. Structural prior: We assign higher priority to the SSM backbone, which possesses O(n) linear efficiency and captures the vast majority of the skeletal context, ensuring the model’s efficiency foundation in large-scale supercell computations. Physical priors: Attention is defined as an “on-demand” auxiliary correction. In magnetic prediction, this means that the model first attempts to capture conventional spin correlations through scanning (handled by Mamba), and only invokes the attention branch for global correction when encountering complex long-range dependencies or extreme lattice distortions. As the training epochs progress, the model is able to spontaneously adjust this weight ratio based on the topological complexity of dataset-specific doping systems, such as those in the CMR dataset, demonstrating improved task adaptability.

### 4.4. Analysis of Computational Complexity and Practical Computational Efficiency

In the design of graph foundation models, the trade-off between theoretical asymptotic complexity and empirical computational overhead is central to determining whether an architecture can be successfully implemented. As mentioned earlier, the Mamba-2 branch achieves strict linear complexity through a hardware-aware SSD algorithm. However, the introduction of multi-head attention branches raises a critical theoretical question: since global attention is retained, is the entire model still trapped in an O(n^2^) quagmire? From a purely asymptotic complexity perspective, although the theoretical complexity includes an O(n^2^) term due to the retention of the global attention branch, the actual computational constant is extremely small because we have performed extreme dimensionality reduction (φ ≥ 4) and layer reduction on the attention branch. At the scale of nodes used in the experiments (n ≤ 1400),the growth curves for memory usage and computation time of this model almost coincide with those of the pure linear model (Graph-Mamba), indicating that its empirical scaling is near-linear within the engineering-relevant node counts studied here, although the formal asymptotic complexity of the full architecture remains O(n^2^) (see Appendix E).

To address concerns that this dimension-reduction design may compromise the semantic integrity of non-Euclidean geometry, this paper introduces the concept of “Effective Representation Density”. We believe that when processing physical graph signals, the “focus” of features is far more critical than mere “high dimensionality”. Since the Mamba-2 backbone already carries the vast majority of the skeletal context in full dimensions, the task of the Attention branch is no longer to perform full-scale representation, but rather to establish precise “topological indexing” within a low-dimensional subspace. Mathematically, establishing topological shortcuts requires only determining binary logical relationships between nodes, rather than recording their complex full-scale physical states. This decoupled design generates an “information bottleneck” effect: it forces the auxiliary branch to filter out uninformative feature fluctuations and instead focus, with higher representation density, on the sparse, low-rank dependency patterns relevant to the target property. This collaborative mode—where the main branch is responsible for “thickness” (rich fundamental characteristic manifolds) and the auxiliary branch for “span” (precise non-Euclidean topological shortcuts)—is precisely the key mechanism underlying the model’s ability to fully preserve high-dimensional non-Euclidean topological semantics while maintaining *O*(*n*) empirical efficiency.

This is not a simple attempt at linearization, but rather a pragmatic engineering trade-off designed to balance ‘global routing capabilities’ with ‘high-throughput inference requirements’. We provide an in-depth analysis of this architecture’s ‘pragmatic efficiency’ across the following three levels:

Constant-Order Performance Degradation Resulting from Architectural Decoupling: In traditional architectures such as GraphGPS, the self-attention mechanism serves as the sole backbone for feature routing, requiring multiple layers to be stacked across the full dimension, which results in extremely large quadratic terms. In this architecture, the burden of long-range memory has been taken over by Mamba-2, which has an *O*(*n*) complexity, and the attention branch has been successfully downgraded to a lightweight “topological regularizer”. We employ an extreme dimension compression strategy: projecting the latent feature dimension of the attention branch onto *d_attn_* = *d*/ϕ (where the compression ratio ϕ ≥ 4) and reducing the number of network layers by 50%. At this point, the computational complexity of a single-layer attention is optimized to:

$${\mathrm{Complexity}}_{\mathrm{Attn}}\approx O\left(n^{2}\cdot \frac{d}{\phi }\right)$$

When processing supercells containing thousands of atoms, the quadratic term’s GPU memory usage and computational time during the actual forward pass are minimized due to the extreme dilution of the constant term 1/ϕ, resulting in values far lower than those of a standard Graph Transformer.

Training Dynamics and Convergence Acceleration: The true metric for evaluating model efficiency is the total number of floating-point operations (FLOPs) required to reach convergence. Pure Graph Transformers, lacking inductive biases for atomic local topologies, often require a blind search within a vast hypothesis space during the early training stages, resulting in slow convergence of the loss function [53]. In contrast, our model benefits from the magnetocluster-aware ordering strategy proposed, which provides the model with a high-quality initial feature manifold. This powerful topological guidance smooths the loss landscape. In experimental evaluations, our model achieved an approximately 30–40% faster convergence compared to the pure Transformer baseline. This implies that although each step incurs a minor *O*(*n*^2^) overhead, the overall training duration (wall-clock time) is reduced compared to traditional architectures due to the substantial decrease in the total number of iterations.

In summary, although MagCluster-GMAF does not achieve absolute linearity at the theoretical upper bound, it essentially represents a pragmatic engineering trade-off tailored for large-scale materials computations. We trade a computational burden of O(n^2^) computational burden to gain the precise global routing capabilities that the Mamba series of models lack when dealing with the non-Euclidean topology of two-dimensional materials. Compared to methods that forcibly reduce complexity by sacrificing graph integrity (such as node sampling or sparsification), this architecture introduces controlled quadratic auxiliary branches, thereby compensating for the non-Euclidean semantic information lost during graph dimensionality reduction in purely linear sequence models.

### 4.5. Physics-Informed Design Choices and Their Scope

Three design choices embed physical prior knowledge from dilute-magnetic-doping physics. Here, we state each of them explicitly and map it onto the nanoscale magnetic behavior it is intended to capture, together with the quantitative scope of the claim.

Surface defects: defect-type and dopant conditioning. The defect type (adsorption-site index or interstitial site), the dopant identity, and the formation energy enter the model as graph-level attributes; they are encoded by a two-layer MLP and injected as per-sample conditioning of the readout (Methods), while the dopant identity additionally enters the node features as a binary indicator. Adsorption configurations in our dataset (e.g., the Mn adsorbate on Cr2I6 in Table 7) are precisely such surface-defect structures, so the model is explicitly conditioned on the defect-engineering variables that experiments in this field control. The case studies in Table 7 show that configurations whose moment is dominated by a single magnetic dopant at the surface are indeed the most reliably predicted ones.

Spatial boundary effects: the two-branch architecture. A local branch and a global branch (Mamba-2 in parallel with multi-head attention over a cluster-aware ordered node sequence) coexist because magnetism in doped two-dimensional materials involves both local hybridization around the defect and spatially extended correlations across the supercell; defect–host hybridization and defect–image interactions decay over length scales that can span a large fraction of the cell, and their interplay is mediated by the lattice topology encoded in the supercell graph (5.0 Å cutoff, periodic boundaries). The global branch provides the architectural capacity to propagate information across the entire structure, complementing the local branch. This is a design analogy—a structural hypothesis about which inductive biases are useful—and not a claim that the branches identify the respective physical mechanisms (see the interpretability statement in the Conclusions).

Isolated-atom magnetic priors: the reference state. The three explicit magnetic descriptors—the spin-only moment of the isolated neutral atom, the spin-state identifier, and the magnetic-strength classification (Table 2)—import textbook knowledge about the isolated magnetic atom, the reference state around which defect-induced moments in doped systems are conventionally discussed. Their quantitative role is small and transparent: the feature-group ablation (Table 6) shows that removing all explicit magnetic descriptors costs only 2.1% MAE (0.2739 versus 0.2683 μB), and chemistry-only features retain R^2^ = 0.697. The priors thus act as leakage-free anchors, while the nontrivial effects of moment quenching and antiferromagnetic cancellation are learned from the lattice topology itself.

Scope: The training targets are 0 K spin-polarized DFT moments of relaxed periodic supercells. Finite-temperature variations—thermodynamic spin fluctuations, magnetic-anisotropy barriers, and temperature-dependent ordering—are therefore not part of the learned mapping, and we do not claim otherwise; extending the framework to finite-temperature magnetism (e.g., learning from temperature-resolved data) is identified as future work. Within its scope, the model is conditioned on exactly the variables that defect engineering controls, as the case studies in Table 7 illustrate.

## 5. Conclusions and Outlook

In this study, we propose MagCluster-GMAF, a physics-inspired hybrid graph architecture specifically designed to accurately predict the macroscopic magnetism of doped two-dimensional nanomaterials. By transforming complex doped supercells into property graphs infused with deep physical descriptors, our method relates atomic-level chemical descriptors to macroscopic magnetic moments. This framework establishes a favorable trade-off boundary between computational efficiency and topological expressiveness, alleviating the computational bottleneck of traditional global graph attention networks *O*(*n*^2^), but also addressing the semantic fragmentation inherent in purely state-space models such as Graph-Mamba. A key design element of our platform is the dual-branch coordination mechanism. The lightweight attention branch acts as a topological regularizer: fusion-ratio ablations (Table 5) show that introducing this branch reduces the MAE from 0.3054 to 0.2729, and the learnable fusion weights spontaneously converge to approximately 75/25 during end-to-end training, indicating that the branch serves as a residual topological compensation module rather than a general-purpose feature extractor. Furthermore, SHAP attribution analysis confirms that the model’s predictions are strongly anchored by intrinsic magnetic descriptors, such as the number of unpaired electrons, and are modulated by chemical environment features, such as electronegativity. The success of the Mamba-2 backbone relies heavily on our “magnetic cluster-aware ordering” strategy, which fuses structural degree, feature similarity, and regularization noise (40/40/20). This physics-inspired flattening mechanism acts as an implicit soft clustering mechanism, improving sequence-level semantic coherence and enabling short-range convolution kernels to aggregate locally coherent features. The introduction of dynamic noise provides stochastic regularization that prevents overfitting to absolute one-dimensional positions and ensures robust implicit structural encoding. During end-to-end training, the dynamic decoupling of roles between the two branches naturally converges to a steady state: Mamba-2 carries the primary representation load, while the attention branch is streamlined into a highly specialized topological regularization fallback module. This synergistic decoupling enables the architecture to exhibit near-linear empirical scaling on large graphs at the scales studied, substantially alleviating—though not formally eliminating—the memory and computational latency barriers associated with global graph attention.

We distinguish two levels of explanation: model interpretability, which concerns how a learned predictor uses its inputs (e.g., feature attributions), and physical-mechanism explanation, which concerns the actual microscopic interactions in the material. Only the former is claimed in this work: the interpretability established here is limited to feature-level attribution (SHAP); the attention weights are analyzed only as model-internal diagnostics. A physically grounded validation of attention mechanisms, for example, against DFT-derived exchange pathways, is left for future work.

From an application perspective, the trained model can serve as an inexpensive pre-filter that narrows a large space of doped-structure candidates to a shortlist for targeted DFT validation, as illustrated by the case studies and ranking fidelity on held-out structures (Table 7; Figure 2). Extending this screening loop to unexplored composition–defect combinations—and validating the high-moment candidates it surfaces by full DFT calculations—is the immediate next step of this work.

The exceptional computational efficiency and generalization capabilities of the MagCluster-GMAF architecture provide a versatile, general-purpose foundation for future high-throughput screening of magnetic nanomaterials. Although the current model focuses on the regression of macroscopic total magnetic moments, this framework can be extended to multi-task learning to simultaneously predict localized atomic magnetic moment distributions, density of states (DOS), and magnetic anisotropy energy. Furthermore, the introduction of spatiotemporal graph modeling can elucidate spin perturbations in complex co-doped or high-entropy two-dimensional systems under the influence of temperature fields and lattice dynamics. Ultimately, deploying this linearly efficient architecture as a pre-trained foundation model for high-throughput screening is expected to accelerate the ab initio design of next-generation spintronic devices, thereby bridging the gap between rigorous physical mechanisms and engineering scalability.

## Figures and Tables

**Table 1:** Overview of baseline models. The selected baselines encompass various evolutionary paradigms, including exact global attention, kernel approximation, sparse attention, expanding graph attention, and pure state-space models.

Baseline Model	Attention Mechanism	Complexity	Key Innovation
**GPS + Transformer** [21]	Global Self-Attention	O(n^2^)	Expression Capacity Upper Limit Benchmark
**GPS + Performer** [22]	Random Feature Mapping	O(n√n)	Nuclear Approximate Linearization
**GPS + BigBird** [23]	Sparse Attention	O(n log n)	Window + Random + Global Node
**Exphormer** [24]	Extended Graph Attention	O(n)	Virtual node + retention of spectral characteristics
**Graph-Mamba**	Pure Mamba Architecture	O(n)	Degree Sorting Graph serialization

**Table 2:** Node-feature engineering: definitions, origins, and normalization of the 18 atomic descriptors used for total-moment prediction. Note. “Origin” indicates the source of each descriptor: P = periodic-table position; H = Hund’s-rule ground-state electron configuration of the isolated neutral atom; T = textbook magnetic classification of the elements; C = compositional metadata of the input structure file (dopant identity); S = static geometric/chemical constant (Pauling electronegativity, covalent radius). All continuous descriptors are min–max normalized to [0, 1] with fixed constants determined by their theoretical ranges, so that every feature is a deterministic function of the chemical element symbol (and for No. 3, of the dopant identity supplied with the input structure). None of the 18 features was derived from the DFT calculations used to produce the target total magnetic moments, and all 18 descriptors were available prior to any simulation.

No.	Category	Feature	Definition/Description	Origin	Normalization/Range	DFT-Derived?
1	Basic structural & compositional	Atomic number	Atomic number *Z* of the node’s element	P	*Z*/83 ∈ [0, 1]	No
2	Basic structural & compositional	Group number	Periodic-table group of the node’s element	P	group/80 ∈ [0, 1]	No
3	Basic structural & compositional	Dopant indicator	1 if the atom is the dopant species, 0 otherwise; dopant identity is read from the input structure file	C	binary {0, 1}	No
4	Basic structural & compositional	3*d* transition-metal indicator	1 if 21 ≤ *Z* ≤ 30 (Sc-Zn)	P	binary {0, 1}	No
5	Basic structural & compositional	4*d* transition-metal indicator	1 if 39 ≤ *Z* ≤ 48 (Y-Cd)	P	binary {0, 1}	No
6	Basic structural & compositional	5*d* transition-metal indicator	1 if 72 ≤ *Z* ≤ 80 (Hf-Hg)	P	binary {0, 1}	No
7	Basic structural & compositional	Chalcogen indicator	1 if the element belongs to {S, Se, Te}	P	binary {0, 1}	No
8	Basic structural & compositional	Common 2D-host indicator	1 if the element belongs to {Mo, W, Nb, Ta, Re}	P	binary {0, 1}	No
9	Periodic-table attribute	Period	Period index of the element in the periodic table	P	(period − 1)/8 ∈ [0, 1]	No
10	Periodic-table attribute	Electronegativity	Pauling electronegativity χ of the element	S	(χ − 0.5)/3.5 ∈ [0, 1]	No
11	Periodic-table attribute	Covalent radius	Covalent radius *r* of the element (pm)	S	(*r* − 25)/225 ∈ [0, 1]	No
12	Periodic-table attribute	Orbital block	Valence-orbital block of the element: s = 0, p = 1/3, d = 2/3, f = 1	P	{0, 1/3, 2/3, 1}	No
13	Electronic structure	Unpaired-electron count (*n*_unpaired)	Number of unpaired electrons in the Hund’s-rule ground-state electron configuration of the isolated neutral atom	H	*n*_unpaired/14 ∈ [0, 1]	No
14	Electronic structure	*d*-orbital occupation (*n*_d)	Number of electrons in the *d* orbitals of the isolated neutral atom (ground-state configuration)	H	*n*_d/10 ∈ [0, 1]	No
15	Electronic structure	*f*-orbital occupation (*n*_f)	Number of electrons in the *f* orbitals of the isolated neutral atom (ground-state configuration)	H	*n*_f/14 ∈ [0, 1]	No
16	Magnetic prior	Theoretical magnetic moment (μ_iso)	Spin-only magnetic moment of the isolated neutral atom: μ_iso = 2·[*S*(*S* + 1)]^(1/2) μ_B, with *S* = *n*_unpaired/2	H	μ_iso/15 ∈ [0, 1]	No
17	Magnetic prior	Spin-state identifier	1 if *n*_unpaired > 0 (spin-carrying atom), 0 otherwise	H	binary {0, 1}	No
18	Magnetic prior	Magnetic-strength class	Three-level textbook classification: strong (Fe, Co, Ni, Gd, Dy, Ho, Er) = 1.0; intermediate (Cr, Mn, V, Ti, Sc) = 0.5; weak/non-magnetic = 0.0	T	{0, 0.5, 1}	No

**Table 3:** Performance comparison of various graph neural network architectures for the magnetic property prediction of 2D nanomaterials. Best results are colored in first, second, third. Values are reported as the mean ± standard deviation over five independent runs (seeds 0–4).

Model	MAE ↓	*R*^2^ ↑	MSE ↓	SpearmanR
**GPS + Transformer**	0.2965 ± 0.0018	0.6323 ± 0.0013	0.3828 ± 0.0023	0.5264 ± 0.0012
**GPS + Performer**	0.2841 ± 0.0023	0.7414 ± 0.0024	0.3364 ± 0.0022	0.5591 ± 0.0032
**GPS + BigBird**	0.2741 ± 0.0016	0.6807 ± 0.0007	0.3076 ± 0.0014	0.5188 ± 0.0019
**Exphormer**	0.2755 ± 0.0021	0.7034 ± 0.0015	0.2856 ± 0.0015	0.5423 ± 0.0041
**Graph-Mamba**	0.2739 ± 0.0012	0.7252 ± 0.0021	0.3561 ± 0.0006	0.5039 ± 0.0023
**MagCluster-GMAF**	0.2683 ± 0.0027	0.7152 ± 0.0008	0.2747 ± 0.0018	0.5500 ± 0.0015

**Table 4:** Full results of the ordering strategy ablation study. The comprehensive strategy achieved the best performance on both the magnetic property prediction and peptide function classification tasks, verifying the cross-domain robustness of the “structured semantic clustering + stochastic perturbation” strategy.

Ordering Strategy	MAE ↓ (CMR)	AP ↑ (Peptides-Func)
No strategy	0.30831	0.6581
Random sort	0.29625	0.6691
Based on node degree sort	0.29374	0.6834
Feature similarity sort	0.28746	0.6859
Multi-criteria ranking	0.27302	0.6881

**Table 5:** Ablation data of branch fusion ratios. The 60/40 ratio achieves performance extremum on both tasks; an excessively high proportion of the Attention branch leads to information redundancy and potential overfitting.

Branch Fusion Ratio	MAE ↓ (CMR)	AP ↑ (Peptides-Func)
mamba2 (100%) + Ts (0%)	0.3054	0.6542
mamba2 (90%) + Ts (10%)	0.2873	0.6583
mamba2 (80%) + Ts (20%)	0.2892	0.6679
mamba2 (70%) + Ts (30%)	0.2856	0.6746
mamba2 (60%) + Ts (40%)	0.2729	0.6881
mamba2 (50%) + Ts (50%)	0.2784	0.6754

**Table 6:** Feature-group ablation for total-moment prediction.

Feature Group	Dim	MAE↓ (μB)	MSE	R^2^	SpearmanR	ΔMAE vs. full
F4: full feature set	18	0.2683	0.2747	0.7152	0.5500	—
F2: w/o all explicit magnetic descriptors	15	0.2739	0.2815	0.7087	0.5401	+2.1%
F3: isolated-atom magnetic priors only	15	0.2846	0.2791	0.7126	0.5298	+6.1%
F1: structural/chemical descriptors only	12	0.2895	0.2918	0.6973	0.5174	+7.9%

**Table 7:** Representative case studies from the held-out test set. Note. Configurations are drawn from the held-out test split; predictions are single-model outputs. The DFT moments are the total magnetic moments of the DFT-relaxed structures. The average absolute error across the six cases (0.28 μB) is consistent with the test MAE of 0.2683 μB (Table 3).

Configuration	Dopant/Defect Setting	DFT Moment (μB)	Predicted (μB)	Abs. Error (μB)
Cr2I6_Mn_ads1	Mn adsorbate (ads1 site) on Cr2I6	+31.00	+31.42	0.42
MoS2_Mn_int2	Mn interstitial (int2 site)	+7.00	+6.77	0.23
Te2W_Fe_ads2	Fe adsorbate (ads2 site)	+4.00	+3.74	0.26
NbS2_As_ads0	As adsorbate (ads0 site)	+4.00	+4.33	0.33
MoS2_Zn_ads1	Zn adsorbate (ads1 site)	0.00	−0.16	0.16
NbSe2_S_int3	S interstitial (int3 site)	−3.00	−3.29	0.29

## Data Availability

All datasets used in this study, including the doped two-dimensional magnetic materials dataset, are available from the corresponding references. The processed dataset supporting the findings of this work is available from the corresponding authors upon reasonable request. The custom code for model training and evaluation is available from the corresponding authors upon reasonable request.

## Appendix A. About the Dataset

To ensure the comprehensiveness and generalizability of our model performance evaluation, we conducted extensive empirical evaluations on standard benchmark datasets across multiple interdisciplinary fields. As shown in Table A1, we selected five graph datasets spanning materials science, computational biology, computer vision, and software network topology. These five datasets range from micro-scale physical constraints (CMR, Peptides) to macro-scale scale-free networks (MALNET), fully demonstrating that our multi-hybrid ranking strategy (40/40/20) is not only applicable to homogeneous graphs, but is also robust against heterogeneous noisy graphs. These datasets exhibit significant differences in graph scale, topological heterogeneity, and task types, with a particular focus on testing the model’s ability to capture long-range graph context.

Dataset of Doped Two-Dimensional Nanomaterials (CMR Impurities): This dataset originates from the Computational Materials Repository (CMR). This dataset includes approximately 17,000 doped two-dimensional materials collected through a high-throughput first-principles (DFT) calculation system.

Peptide Molecular Graph Datasets (Peptides-Func & Peptides-Struct): Both datasets originate from the Long-Range Graph Benchmark (LRGB) and contain 15,535 peptide molecular graphs, with an average of 150.9 nodes.

Image Superpixel Datasets (PascalVOC-SP & COCO-SP): Raw images are converted into superpixel graphs using the SLIC algorithm, with an average of 479.4 nodes.

Malware Function Call Graphs (MalNet-Tiny): This dataset consists of function call graphs extracted from Android APKs. With an average of 1410.3 nodes, it is a critical dataset for validating a model’s scalability at large scales. In this benchmark version, the original node and edge features have been removed, retaining only structural information (Local Degree Profile), thereby forcing the model to rely entirely on graph topology for malware classification.

**Table A1:** Overview of cross-domain benchmark datasets. The table summarizes the statistical information and task attributes of the five benchmark datasets employed in this study, spanning diverse domains including materials science (CMR Impurities), computational biology (Peptides-Func and Peptides-Struct), computer vision (VOCSuperpixels), and malware detection (MALNET-TINY). These datasets differ in graph scale, topological heterogeneity, and task type, aiming to comprehensively evaluate the model’s long-range context modeling capability and cross-domain generalization performance.

Dataset	Task Type	Graph Size	Metric	Domain
**CMR Impurities**	Regression	Atomic-level lattice diagram	MAE ↓	Materials science
**Peptides-Func**	Multi-Label Classification	Graph of 15,535 peptide molecules	AP ↑	Computational biology
**Peptides-Struct**	Multi-Objective Regression	Graph of 15,535 peptide molecules	MAE ↓	Computational biology
**VOCSuperpixels**	Node Classification	Superpixel segmentation image	Macro-F1 ↑	Computer vision
**MALNET-TINY**	Image Classification	Average number of nodes > 1000	Accuracy ↑	Malware detection

## Appendix B. Additional Experimental Results

To validate the generality of the MagCluster-GMAF architecture across different topological heterogeneities and task types, this study conducted extended experiments on four standard graph learning benchmark datasets from non-materials domains: Peptides-Func, Peptides-Struct (computational biology), VOCSuperpixels (computer vision), and MalNet-Tiny (software analysis). As shown in Table A2, the core rationale for selecting these datasets lies in their shared requirement for modeling significant long-range context, which rigorously tests the model’s pure architectural expressiveness when operating without physical priors.

**Table A2:** Performance comparison on cross-domain standard graph benchmarks. MagCluster-GMAF achieves the best or near-best results among the evaluated baselines on diverse datasets spanning biology, vision, and software analysis, demonstrating exceptional generalization capability and architectural universality. Best results are colored in first, second, third. Values are reported as mean ± standard deviation over five independent runs (seeds 0–4).

Model	Peptides-Func	Peptides-Struct	VOCSuperpixels	MALNET-TINY
	AP ↑	MAE ↓	F1 ↑	Accuracy ↑
**GPS + Transformer**	0.6573 ± 0.0034	0.2842 ± 0.0130	0.3774 ± 0.0150	0.9223 ± 0.0065
**GPS + Performer**	0.6475 ± 0.0043	0.2558 ± 0.0012	0.3823 ± 0.0148	0.9264 ± 0.0078
**GPS + BigBird**	0.6139 ± 0.0064	0.2510 ± 0.0015	0.2622 ± 0.0008	0.9234 ± 0.0034
**Exphormer**	0.6358 ± 0.0075	0.2512 ± 0.0025	0.3430 ± 0.0108	0.9422 ± 0.0024
**Graph-Mamba**	0.6739 ± 0.0043	0.2478 ± 0.0016	0.3761 ± 0.0101	0.9340 ± 0.0027
**MagCluster-GMAF**	0.688 ± 0.0032	0.2494 ± 0.0017	0.3960 ± 0.0175	0.9375 ± 0.0053

In summary, MagCluster-GMAF demonstrated competitive performance across diverse fields such as computational biology, computer vision, and malware detection, achieving the best or near-best results among the evaluated baselines on multiple datasets. Experiments confirm that the lightweight attention branch effectively functions as a “topological regularizer”, compensating for information loss that may occur during one-dimensional sequence scanning when processing complex non-Euclidean structures. Furthermore, thanks to the linear efficiency of the backbone network *O*(*n*), this architecture successfully avoids the memory explosion issues associated with traditional Transformers when handling large-scale graph tasks with over a thousand nodes (such as MalNet-Tiny), reaching the favorable trade-off boundary of expressiveness while maintaining extremely low computational overhead. The “40/40/20” hybrid ordering strategy proposed by this architecture also demonstrates exceptional cross-domain robustness, ensuring high-quality transmission of feature manifolds across different semantic contexts by preserving structural and semantic continuity during dimensionality reduction.

## Appendix C. Mathematical Derivation and Proof of State Space Duality (SSD)

### Appendix C.1. Basic Form of Structured State-Space Models (SSM)

In the Mamba-2 architecture, the core operator is a linear time-varying system (LTV), and its discretized state space equation is defined as follows:

*h_t_* = *A_t_h_t_*_−1_ + *B_t_x_t_*

*y_t_* = *C_t_h_t_*

In this context, *x_t_*,*y_t_* denote the input and output sequences, respectively, and h*_t_* ∈ R*^N^* is the hidden state. In the simplified setting of Mamba-2, it is assumed that *A_t_* is scalar-valued and satisfies the selective scanning constraint [54].

### Appendix C.2. Representation of Semiseparable Matrices

The recursive process of state-space equations can be fully expressed as a matrix mapping *y* = *Mx*. For a causal system, the matrix M is a lower-triangular matrix, and its elements *M_i_*_,*j*_ are defined as:

$$M_{i,j}=\{\begin{array}{c} C_{i}A_{i:j+1}B_{j}\mathrm{ifi}>j\\ C_{i}B_{i}\mathrm{ifi}=j\\ 0\mathrm{ifi}<j \end{array}$$

In particular, *A_i_*_:*j*_ = *A_i_*_−1_*A_i_*_−2_ … *A_j_* represents the cumulative product of state transitions. A matrix with this structure is called a 1 × 1 semiseparable matrix.

### Appendix C.3. Derivation of the State Space Duality (SSD) Theorem

The core of SSD lies in proving that the SSM operator described above is equivalent to a structured attention mechanism. We performed a low-rank decomposition on the lower triangular part. Let L be the matrix containing all *C_i_* information terms, and let R be the matrix containing all *B_j_* information terms. Under the influence of the state transition operator A, matrix M can be written as:

$$M=L\circ \tilde{M}\cdot R^{⊤}$$

where ∘ denotes the Hadamard product, and $\tilde{M}$ is a matrix composed of the repeated terms A_i:j_. In the standard attention mechanism, y = softmax(QK^⊤^)V. Under the SSD theory, we can regard C_t_ as the query, B_t_ as the key, and x_t_ as the value. Linear View (Recurrent View): Recursive computation via hidden state h*_t_*, with a complexity of *O*(*L* ⋅ *N*). Dual View (Matrix View): Directly computes the product of matrix M and the input vector, which manifests as a restricted form of linear attention with an exponential decay mask:

*Y* = (*QK*^⊤^∘*S*)*V*

Here, S is the decay operator determined by the state transition matrix A.

## Appendix D. Precise Definitions and Provenance of Atomic Descriptors

All node features used in this work are static functions of the chemical element symbol alone and are computed from closed-form lookup tables prior to any simulation. No feature is derived, directly or indirectly, from the density functional theory (DFT) calculations that generate the prediction target (the total magnetic moment). In particular, the three magnetic-prior descriptors are defined as follows:

(i) Number of unpaired electrons (n_unpaired_). The number of unpaired electrons in the ground-state electron configuration of the isolated neutral atom, determined by Hund’s rules from the periodic-table position of the element.

(ii) Theoretical magnetic moment estimate (μ_iso). The spin-only magnetic moment of the isolated neutral atom is:

*μ*_*iso* = 2 · [*S*(*S* + 1)](1/2)*μ*_*B*,*with S* = *n*_*unpaired*/2

where S is the total spin quantum number implied by Hund’s-rule ground-state filling. This quantity depends only on the atomic electron configuration and involves no information from any DFT calculation.

(iii) Spin-state identifier. A binary indicator, 1 if n_unpaired > 0 and 0 otherwise, encoding whether the isolated atom carries a spin moment at all.

(iv) Magnetic-strength classification. A three-level categorical label (strong: 1.0; intermediate: 0.5; weak/non-magnetic: 0.0) assigned according to the well-established room-temperature ferromagnetic/antiferromagnetic character of the elements (e.g., Fe, Co, Ni, Gd classified as strong), as documented in standard textbooks. This classification is not derived from measured or computed data associated with the materials in our dataset.

All continuous descriptors are min–max normalized to [0, 1] using fixed constants determined by their theoretical ranges (e.g., n_unpaired/14,μ_iso/15μ_B) and are therefore identical across all samples containing the same element. A complete provenance audit of all 18 node features—definition, origin, and DFT-independence—is provided in Table 2.

## Appendix E. Complexity Analysis of the Hybrid Architecture

### Appendix E.1. Component-Wise Asymptotic Complexity

Let n denote the number of nodes in a doped supercell, d is the latent hidden dimension, φ ≥ 4 is the compression ratio of the attention branch (so that its effective feature dimension is d_a_ = d/φ), and L_m_, L_a_ are the numbers of Mamba-2 and the attention layers, respectively. In our configuration, the attention branch uses roughly half the depth of the Mamba-2 backbone (L_a_≈L_m_/2) and accounts for less than 0.3% of the total parameters. Table A3 summarizes the per-branch asymptotic costs.

**Table A3:** Component-wise asymptotic complexity of MagCluster-GMAF.

Component	Core Operation	Asymptotic Cost	Constants in This Work
Mamba-2 branch (backbone)	Selective state-space scan over the serialized sequence	O(*L_m_* · *n* · *d*)—linear in *n*	Full hidden dimension *d*; hardware-aware parallel scan
Attention branch (auxiliary)	Scaled dot-product attention (QK^T and A·V)	O(*L_a_* · *n*^2^·*d_a_*) = O(*L_a_* · *n*^2^·*d*/*φ*)—quadratic in *n*	Feature dimension reduced by *φ* ≥ 4; layer count ≈ backbone/2; <0.3% of parameters
Dynamic fusion	Learnable weighted superposition of branch outputs	O(*n* · *d*)	Two scalar gates
Readout & prediction head	Global mean pooling + MLP	O(*n* · *d*)	—

### Appendix E.2. Formal Complexity of the Full Architecture

Summing the branch costs, the total computational cost is:

*C*(*n*) = *L_m_* · *n* · *d* + *L_a_* · *n*^2^·*d*/*φ* + *O*(*n* · *d*) = *a* · *n* + *b* · *n*^2^,*witha* = *L_m_* · *d*,*b* = *L_a_* · *d*/*φ*.

We therefore state explicitly that the formal asymptotic complexity of the full hybrid architecture is O(n^2^) as n → ∞, inherited from the global attention branch; the architecture is not O(n) in the asymptotic sense. What the co-design achieves is (i) a reduction in the effective constant of the quadratic term by the compression ratio φ and the halved attention depth, and (ii) the preservation of an O(n) backbone that dominates the cost within the practically relevant regime.

### Appendix E.3. Empirical Scaling in the Studied Regime

For *C*(*n*) = *a* · *n* + *b* · *n*^2^, the linear term dominates whenever *n*≪*a*/*b*. With φ ≥ 4 and the halved attention depth, the empirical measurements in Figure 7 show that both the peak GPU memory and time per epoch of MagCluster-GMAF closely follow the pure-Mamba linear baseline for 100 ≤ n ≤ 1400, confirming that the node counts studied in this work lie within the linearly dominated range. The quadratic term would become dominant only at node counts well beyond this benchmark range; at such scales, however, the dense O(n^2^) memory footprint of any full global-attention baseline would itself become prohibitive, so the practical advantage of the hybrid design persists. We therefore describe the scalability of the architecture as empirically near-linear at the studied scales, and refrain from claiming formal linear-time complexity.
